# Senescent immune cells in the tumor microenvironment: emerging insights into cancer immunotherapy resistance

**DOI:** 10.3389/fimmu.2025.1656733

**Published:** 2025-10-17

**Authors:** Duolun Gao, Peiyan Kan, Yanjie He, Siyu Sun, Lei Tang, Fan Yang

**Affiliations:** ^1^ Department of Gastroenterology, Engineering Research Center of Ministry of Education for Minimally Invasive Gastrointestinal Endoscopic Techniques, Shengjing Hospital of China Medical University, Shenyang, Liaoning, China; ^2^ Department of Surgery, New York University School of Medicine and New York University -Langone Medical Center, New York, NY, United States; ^3^ Department of Endocrinology, Shengjing Hospital of China Medical University, Shenyang, Liaoning, China

**Keywords:** tumor microenvironment, immune cell senescence, immunosuppression, cancer immunotherapy, anti-aging therapy, cancer treatment strategies

## Abstract

Cancer remains a leading cause of mortality worldwide, with rising incidence and death rates continuing to rise. While conventional treatments such as surgery, radiotherapy, and chemotherapy form the backbone of cancer care, they are often limited by adverse effects, recurrence risk, and incomplete tumor eradication. Tumor immunotherapy—particularly immune checkpoint inhibitors and chimeric antigen receptor (CAR) T cell therapy—has emerged as a transformative approach by activating and reprogramming anti-tumor immune responses. Despite these advances, significant challenges persist, including limited response rates to checkpoint inhibitors, the immunosuppressive nature of the tumor microenvironment (TME), and resistance mechanisms employed by tumor cells. Growing evidence suggests that immune cell senescence is a critical contributor to TME-driven immunosuppression. Senescent immune cells exhibit functional decline, elevated expression of inhibitory immune checkpoint molecules, and increased secretion of pro-inflammatory cytokines, collectively impairing anti-tumor immunity and reducing the efficacy of immunotherapy. This review highlights the role of immune cell senescence in shaping the immunosuppressive TME and driving resistance to immunotherapy. It further discusses emerging therapeutic strategies that combine immunotherapy with senescence-targeting interventions, aiming to provide novel insights into the development of more effective cancer treatment strategies.

## 1 Introduction

According to recent reports, there were approximately 19.665 million new cases of malignant tumors and 9.737 million cancer-related deaths globally in 2022, with projections estimating up to 35 million new cases by 2050 (1). The lifetime risk of cancer-related death is estimated at approximately 11% for males and 8% for females. Advancements in detection technologies have significantly improved the accuracy of cancer diagnoses, further underscoring cancer’s status as a leading cause of mortality worldwide (2–4). In parallel, population aging and lifestyle changes are expected to drive further increases in cancer incidence and mortality (5). Conventional cancer treatments—including radiotherapy, chemotherapy, and surgical resection—remain foundational but are often insufficient to achieve complete tumor eradication, thereby posing risks of recurrence. Additionally, these therapies are frequently associated with substantial side effects that adversely affect patient quality of life and long-term outcomes (6). For example, platinum-based chemotherapies are known to induce cardiotoxicity and nephrotoxicity, while other agents may cause peripheral neuropathy and a range of gastrointestinal complications (7). Moreover, the risk of tumor recurrence following surgical resection remains a significant concern (8). These limitations underscore the urgent need for more effective and durable therapeutic strategies.

Among emerging modalities, immunotherapy has gained prominence as one of the most promising and clinically impactful approaches in cancer treatment. Tumor immunotherapy—particularly immune checkpoint inhibition—aims to reinvigorate exhausted immune cells, reshape the tumor microenvironment (TME), and enhance the cytotoxic functions of effector T cells. For instance, immune checkpoint inhibitors (ICIs) targeting programmed cell death protein 1 and its ligand (PD-1/PD-L1), as well as cytotoxic T lymphocyte-associated protein 4 (CTLA-4), have demonstrated substantial clinical benefits in malignancies such as melanoma and non-small cell lung cancer (NSCLC), significantly improving overall survival (9). Chimeric antigen receptor T cell (CAR-T) therapy has also shown remarkable success in hematologic malignancies, such as acute lymphoblastic leukemia. However, its efficacy in solid tumors remains limited due to a range of barriers, although it continues to represent a breakthrough in cancer immunotherapy (10). Despite these advances, immunotherapy faces considerable challenges. Response rates to ICIs remain below 20% in many cancer types; some tumors exhibit an “immune desert” phenotype with poor immune cell infiltration, and tumor cells often develop resistance through multiple mechanisms, ultimately reducing treatment efficacy (11–13). Within the TME, immune cell senescence has emerged as a key contributor to immune dysfunction. Senescent immune cells typically exhibit impaired functionality, upregulation of immune checkpoint molecules such as PD-1, and increased secretion of pro-inflammatory cytokines These features not only compromise anti-tumor immunity but also contribute to immunotherapy resistance, making immune senescence an increasingly important focus in cancer research (14, 15).

Immune cell senescence refers to the progressive decline in immune system function triggered by various stimuli, with cellular senescence as a central process. This state is characterized by a reduction in immune cell numbers, impaired effector functions, and diminished responsiveness to pathogens and tumor cells (16). Senescent immune cells exhibit hallmark features such as telomere shortening, oxidative damage, elevated levels of reactive oxygen species (ROS), DNA damage, increased expression of cell cycle regulators (e.g., p16^INK4a^ and p21^CIP1^), and a senescence-associated secretory phenotype (SASP) marked by high levels of pro-inflammatory mediators. These changes collectively limit the cells’ proliferative capacity and responsiveness to immunological challenges. In the TME, senescent immune cells—including T cells lacking expression of the co-stimulatory molecule CD28 (CD28^−^ T cells)—exacerbate immune dysfunction, impairing anti-tumor responses and facilitating immune evasion by malignant cells (17, 18). Accumulating evidence indicates that these senescent cells not only fail to clear tumor cells effectively but may also promote tumor progression and metastasis through the secretion of immunosuppressive and pro-tumorigenic factors (19, 20). Once cancer is established, the presence of senescent immune cells severely compromises anti-tumor immunity, correlating with poor patient prognosis, reduced survival, and limited efficacy of immunotherapy.

This review discusses the mechanistic role of immune cell senescence in shaping an immunosuppressive TME, its contribution to immunotherapy resistance, and the therapeutic potential of combining immunotherapy with senolytic agents. Together, these insights aim to inform the development of novel strategies to enhance the efficacy of cancer immunotherapy.

## 2 Composition of the TME

The TME is a highly dynamic and complex ecosystem composed of malignant cells, diverse stromal and immune cell populations, extracellular matrix (ECM) components, and various bioactive molecules. The interplay among these components profoundly affects tumor initiation, progression, metastasis, and therapeutic responses.

### 2.1 Cellular components of the TME

#### 2.1.1 Immune cells in the TME

The TME hosts innate and adaptive immune cells whose interactions determine tumor fate and treatment outcomes. Innate immune cells—including macrophages, neutrophils, dendritic cells (DCs), and natural killer (NK) cells—exhibit marked plasticity with context-dependent functions. M1 macrophages possess anti-tumor activity, whereas M2 macrophages promote immunosuppression, angiogenesis, and therapy resistance (21–23). Tumor-associated neutrophils polarize into N1 (anti-tumor) or N2 (pro-tumor) phenotypes; N1 reprogramming via interferon gamma (IFN-γ) conditioning has shown therapeutic promise (24). NK cells eliminate tumor cells through cytotoxic mechanisms (25). Myeloid-derived suppressor cells (MDSCs), particularly polymorphonuclear (PMN)-MDSCs and monocyte (M)-MDSCs, inhibit T cell activity via contact-dependent (ROS/peroxynitrite) and independent (NO/arginase-1/cytokines) pathways, thereby promoting tumor progression (26–29). DCs serve as a bridge between innate and adaptive immunity by capturing tumor antigens and activating T cells. Subsets such as classical DC1 (cDC1) and CD103^+^ DCs are particularly important for effective anti-tumor immunity (30–32). Cancer-associated fibroblasts (CAFs) contribute to tumor progression by remodeling ECM, secreting immunosuppressive mediators, and promoting angiogenesis (33, 34).

Within the adaptive immune compartment, T cells are play a central role in anti-tumor immunity. However, chronic antigen exposure and TME stress induce T cells exhaustion, characterized by impaired function and elevated expression of inhibitory receptors such as PD-1, CTLA-4—the primary targets of immune-checkpoint blockade (35–37). Regulatory T cells (Tregs) suppress effector T cell activity and sustain tumor-promoting immune tolerance (37). B cells exhibit dual roles: while capable of antibody production, antigen presentation, and tertiary lymphoid-structure formation, they also acquire regulatory functions or produce pro-tumor antibodies under TME influence, expanding MDSCs and dampening immunity (38–40).The functional states of all immune subsets are shaped by cellular crosstalk and metabolic competition within the TME. A comprehensive understanding of this immune-regulatory network is essential for developing effective cancer immunotherapies.

### 2.2 Non-cellular components of the TME

The non-cellular components of the TME—including soluble factors, the extracellular matrix (ECM), and exosomes—form a complex regulatory network that governs tumor progression and immune responses. Immunosuppressive cytokines, notably transforming growth factor beta (TGF-β), synergize with tumor-derived metabolic products to suppress T and NK cell functions, facilitating immune evasion. In contrast, interleukin (IL)-15 activates the janus kinase (JAK)-signal transducer and activator of transcription (STAT) pathway, enhancing T and NK cell cytotoxicity while mitigating MDSC-mediated immunosuppression (41, 42), thereby remodeling the TME in favor of anti-tumor immunity. Pro-inflammatory cytokines like IFN-γ further augment anti-tumor responses by upregulating MHC expression (43–45). The complement cascade also contributes to immunosuppression, with activation fragments C3a and C5a recruiting and polarizing MDSCs, which subsequently secrete IL-10 and TGF-β to reinforce an immune-inhibitory milieu (46).

TGF-β-driven ECM remodeling, characterized by collagen deposition and matrix stiffening, induces epithelial-mesenchymal transition (EMT), enhances tumor invasion through biomechanical stress, and obstructs effector T cell infiltration (47–50). Exosomes play dual roles in TME regulation. Tumor-derived exosomes carry PD-L1 and immunosuppressive miRNAs that systemically blunt immune activity, while immune cell-derived exosomes activate anti-tumor responses via MHC and co-stimulatory molecule delivery (51–55). Additionally, exosomal long non-coding RNAs (lncRNAs) contribute to immunosuppression by modulating immune checkpoint pathways, promoting M2 macrophage polarization, and suppressing NK and CD8^+^ T-cell activity (56). Collectively, these non-cellular elements orchestrate an immunosuppressive and pro-tumorigenic microenvironment, offering promising targets for biomarker development and innovative therapies such as exosome-based drug delivery systems.

### 2.3 Dynamics of the TME

The TME is a dynamic system wherein cellular and non-cellular components engage in continuous interaction through direct contact and paracrine/autocrine signaling, maintaining a shifting equilibrium. In early tumorigenesis, CD8^+^ T cells predominate, mediating tumor cell elimination via cytotoxic activity. As the tumor advances, the TME shifts toward an immunosuppressive state, enriched with MDSCs and Tregs. These cells suppress anti-tumor immunity by secreting inhibitory cytokines and expressing immune checkpoint molecules such as PD-1/PD-L1, thereby facilitating tumor growth and metastasis (57, 58). Tumor-derived metabolic byproducts, including lactate, further exacerbate immunosuppression and alter the physicochemical properties of the TME (59, 60). In later stages, increased angiogenesis and stromal remodeling further promote tumor dissemination and immune evasion (61).

Gastric adenocarcinoma (GAC) exemplifies TME plasticity, which critically influences tumor progression, immune escape, and therapeutic response (62, 63). IgA^+^ plasma cells dominate the pre-neoplastic mucosa, while stromal cells acquire a myofibroblast phenotype predictive of poor prognosis. As GAC progresses, there is a decline in activated CD8^+^ T cells and a corresponding rise in exhausted CD8^+^ T cells, Tregs, tolerogenic DCs, and pro-angiogenic endothelium. Metastatic niches are further characterized by immunosuppressive myeloid-derived CAFs. Two distinct TME ecotypes have been identified: EC3, enriched in CD4^+^/CD8^+^ T cells, NK cells, and DCs, is associated with responsiveness to ICIs, whereas EC6—marked by stromal expansion and dominance of IgG^+^ plasma cells—is linked to diffuse histology and ICI resistance (62). These findings underscore the importance of stage- and ecotype-tailored immunotherapeutic strategies in GAC.

### 2.4 Spatial heterogeneity of the TME

Spatial heterogeneity within the TME refers to region-specific differences in cellular composition, immune activity, and microenvironmental conditions within a single tumor—most notably between the tumor core and periphery. The tumor core often displays an “immune desert” phenotype, marked by hypoxia, metabolic stress, and accumulation of immunosuppressive factors, which collectively restrict immune cell infiltration and promote immune tolerance (12, 13). In contrast, the tumor periphery typically harbors higher densities of effector immune cells, including CD8^+^ T cells and M1-polarized macrophages (64, 65). CD8^+^ T cells in the periphery secrete chemokine (C-C motif) ligand (CCL)3, CCL4, and CCL5, which recruit macrophages via C-C chemokine receptor type 5 (CCR5) signaling. These macrophages are subsequently polarized into the M1 phenotype by IFN-γ, enhancing CD8^+^ T cell cytotoxicity via inducible nitric oxide synthase (iNOS) and promoting antigen presentation (64). Nevertheless, even in the periphery, immune activity is shaped by local immunosuppressive signals, such as serum amyloid A1/2, C-X-C motif chemokine ligand 6 (CXCL6), creating a dynamic balance between activation and suppression (66). This spatial compartmentalization presents challenges and therapeutic opportunities. Strategies aimed at enhancing cytotoxic T cell infiltration and activity within the tumor core with amplifying M1 macrophage-mediated responses in the periphery, may offer synergistic benefits. Accordingly, detailed insights into spatial TME organization are crucial for optimizing immunotherapeutic design and predicting treatment outcomes. Recent advances in high-dimensional spatial profiling have elucidated the TME architectural complexity. In hepatocellular carcinoma, CO-Detection by indEXing (CODEX) has revealed that vimentin-high macrophages frequently co-localize with Tregs (67). These macrophages secrete IL-1β, which enhances Treg-mediated immunosuppression by promoting IL-10 production and inhibiting CD4^+^ T cell proliferation, thereby facilitating immune evasion and disease progression (67). Targeting this macrophage subset may represent a promising strategy for personalized immunotherapy and prognostic refinement.

### 2.5 Temporal heterogeneity of the TME

The temporal heterogeneity of the TME reflects its dynamic evolution during tumor progression, as described by the three-phase cancer-immunity editing cycle (68). In the early elimination phase, the TME is immunologically “hot,” characterized by strong infiltration of effector T cells and NK cells, high IFN-γ levels, and efficient clearance of immunogenic tumor cells. Over time, the tumor enters an equilibrium phase, where immune pressure selects for clones with reduced immunogenicity, allowing tumor persistence. In the escape phase, the TME becomes immunosuppressive or “cold,” dominated by Tregs, MDSCs, M2 tumor-associated macrophages (TAMs), and elevated levels of IL-10 and TGF-β, facilitating immune evasion and therapy resistance. Notably, cellular senescence in tumor and immune cells critically shapes the immune-resistant TME across these stages.

In cervical cancer, high-risk HPV infection promotes carcinogenesis through sustained expression of E6 and E7 oncoproteins, which inactivate p53 and pRb, triggering oncogenic stress. his stress initially activates a senescence response via p15^INK4b^, p16^INK4a^, and p21^Waf1/Cip1^, establishing a potent tumor-suppressive barrier by halting cell proliferation and inducing SASP-mediated immune recruitment. However, continued E6/E7 expression disrupts senescence pathways, enabling a subset of cells to bypass arrest, attain immortality, and transition into the equilibrium phase. This progression supports a potential therapeutic strategy: re-inducing senescence in cancer cells followed by selective senolytic clearance. While conventional therapies can induce cancer cell senescence, they often simultaneously trigger senescence in TME-resident immune cells. The resultant SASP from senescent tumor and immune cells establishes a paracrine loop that reinforces immunosuppression and undermines anti-tumor immunity. Therefore, the development of strategies that prevent or reverse immune cell senescence in the TME is essential to optimize therapeutic efficacy.

## 3 Immune cell senescence in the TME

Aging is characterized by systemic, time-dependent immune deterioration, including thymic involution, reduced populations of naïve immune cells, chronic inflammation, and impaired antigen responsiveness (69). In contrast, immune cell senescence is a stimulus-induced state of irreversible cell cycle arrest, telomere attrition, and a SASP (70). This condition contributes to immune decline by promoting pro-inflammatory cytokine release and loss of cellular functionality. Notably, immune cell senescence can be induced within the TME independently of chronological age, playing a key role in shaping an immunosuppressive milieu.

### 3.1 Induction mechanisms of immune cell senescence

Immune cell senescence can arise through three primary mechanisms: intrinsic senescence, therapy-induced senescence, and TME stress-induced senescence, depending on the nature of the initiating stimuli.

▪ Intrinsic senescence is largely associated with aging and is driven by genomic instability, oxidative stress, and progressive telomere shortening. Over time, accumulated mutations impair DNA repair mechanisms, while excessive ROS generated during metabolism induce oxidative damage. Telomere attrition from repeated cell divisions ultimately triggers senescence signaling pathways, leading to immune dysfunction, chronic inflammation, and increased disease susceptibility (71).
▪ Therapy-induced senescence occurs in response to anticancer treatments such as chemotherapy and radiotherapy, which cause DNA double-strand breaks, ROS overproduction, and activation of inflammatory cascades (72). Chemotherapeutic agents—including topoisomerase inhibitors and alkylating agents—as well as targeted therapies such as cyclin-dependent kinase (CDK) inhibitors, Aurora kinase inhibitors, and epidermal growth factor receptor (EGFR) inhibitors, have been shown to promote immune cells senescence in various cancers, including cervical, colorectal, and breast cancer (73–75).
▪ TME stress-induced senescence is triggered by harsh and metabolically hostile conditions within the TME. Environmental stressors such as hypoxia, nutrient deprivation, oxidative stress, and chronic exposure to inflammatory cytokines released by infiltrating immune and stromal cells activate stress-responsive signaling pathways and transcriptional programs. These ultimately lead to a senescent phenotype in immune cells (76).

Despite their distinct origins, all forms of immune cell senescence converge on common biological features: irreversible cell cycle arrest, impaired effector functions, SASP expression, and immunosuppressive reprogramming.

### 3.2 Characteristics of immune cell senescence

**Figure 1:**
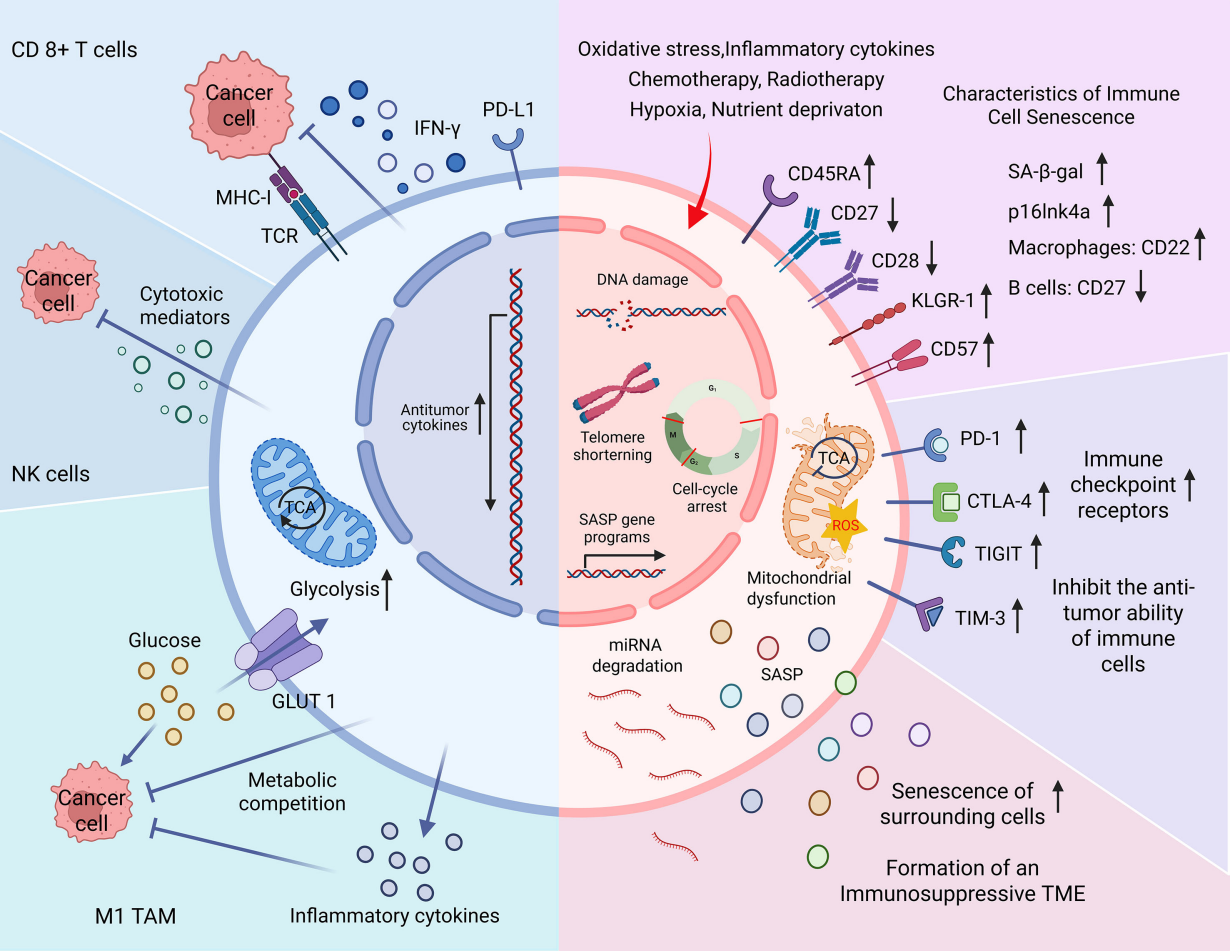
Characteristics of immune cell senescence in the tumor microenvironment (TME). The blue section (left) illustrates the antitumor functions of immune cells. Upon stimulation by major histocompatibility complex class I (MHC-I), CD8^+^ T cells secrete interferon-γ (IFN-γ), perforin, and granzymes to eliminate tumor cells. Natural killer (NK) cells similarly mediate tumor cell lysis by releasing cytotoxic molecules. M1-type tumor-associated macrophages (M1 TAMs) produce pro-inflammatory cytokines and compete with tumor cells for glucose, thereby inhibiting tumor growth through metabolic competition. The pink section (right) depicts the senescence of immune cells triggered by endogenous stress (e.g., oxidative damage), therapy-induced stress (e.g., chemotherapy), and TME-related factors (e.g., hypoxia). Senescent immune cells undergo DNA damage, telomere shortening, and cell cycle arrest. Phenotypic changes include downregulation of CD27 and CD28 and upregulation of CD45RA, CD57, and killer cell lectin-like receptor G1 (KLRG-1) in T cells. Common markers of senescence include increased senescence-associated β-galactosidase (SA-β-gal) activity and p16^Ink4a^ expression. Cell-type–specific alterations include CD22 upregulation in senescent macrophages and CD27 downregulation in B cells. Senescent immune cells also overexpress immune checkpoint receptors, leading to impaired cytotoxic responses. Additional features include mitochondrial dysfunction, microRNA degradation, and the secretion of senescence-associated secretory phenotype (SASP) factors. Together these changes contribute to the development of an immunosuppressive TME and diminished immunotherapeutic efficacy.

Immune cell senescence is characterized by a decline in the number and function of immune cells—particularly CD8^+^ T cells, NK cells, and B cells. This decline results in reduced responsiveness to pathogens, diminished vaccine efficacy, and heightened vulnerability to chronic diseases and cancer (**Figure 1**). In the TME, senescent T cells—especially CD45RA^+^ effector memory T cells re-expressing CD45RA (TEMRA cells)—are commonly observed. These cells typically show reduced expression of co-stimulatory molecules CD27 and CD28, along with increased levels of senescence markers such as CD57, killer cell lectin-like receptor G1 (KLRG-1), and senescence-associated β-galactosidase (77–79). Some terminally differentiated senescent CD8^+^ T cells also partially express natural killer receptors (NKRs), enabling them to respond to antigens independently of T cell receptor (TCR) stimulation (80). In advanced gastric cancer, the expansion of NKR^+^ CD8^+^ T cells has been associated with more aggressive tumor phenotypes (81). Senescence in NK cells is marked by the downregulation of activating receptors and the upregulation of inhibitory receptors, which compromises their cytotoxic capacity against tumor cells (82).

A hallmark of immune senescence is the SASP, which consist of a diverse mix of bioactive molecules, such as pro-inflammatory cytokines (e.g., IL-1β, IL-6, tumor necrosis factor alpha [TNF-α]), chemokines (e.g., CCL2, CXCL8), growth factors (e.g., vascular endothelial growth factor [VEGF], hepatocyte growth factor), and matrix metalloproteinases (MMPs) (83). SASP factors act through autocrine and paracrine mechanisms—supporting tissue repair and clearance of damaged cells on one hand, while on the other hand reshaping the TME and promoting tumor progression in malignancies such as lung and pancreatic cancer (84–87). The dynamic and heterogeneous composition of the SASP underlies its dual function, contributing to either immune stimulation or suppression depending on local microenvironmental cues.

Senescent immune cells also undergo profound epigenetic and metabolic reprogramming. Epigenetically, these cells display altered DNA methylation patterns at specific CpG sites, which impact the expression of genes central to immune function (88). Additionally, histone modification changes and non-coding RNA dysregulation further influence their transcriptomic profiles. Senescent T cells—key effectors in anti-tumor immunity—undergo marked epigenetic remodeling characterized by hypermethylation of gene-silencing CpG sites, repressive histone marks (e.g., H3K27me3, H3K9me3), and hypomethylation in flanking genomic regions (16). Crucially, promoter methylation of genes such as CD27 and SATB1 correlates inversely with their expression, directly impairing T cell functionality. The resultant nuclear landscape, including senescence-associated heterochromatin foci (SAHF), globally elevated chromatin accessibility, and depletion of linker histone H1, severely impairs anti-tumor activity. Moreover, in the CD28^−^ T cell subset, miR-24 overexpression reduces H2AX expression, further impairing DNA damage repair and exacerbating T cell dysfunction (89). To the best of our knowledge, the use of epigenetic-modulating drugs to target immune cell senescence within the TME remains unexplored. This approach holds promise as a potential strategy for enhancing antitumor immunity and warrants further investigation as a future research direction.

Metabolically, senescent immune cells exhibit enhanced glycolytic flux, mitochondrial dysfunction, and elevated ROS levels—indicative of metabolic reprogramming that favors oxidative stress and further reinforces the senescent state (90, 91). Notably, epigenetic and metabolic alterations are interconnected: ROS accumulation can affect the activity of DNA methyltransferases, while aberrant methylation can dysregulate mitochondrial gene expression, forming a self-perpetuating loop of immune dysfunction.

**Figure 2:**
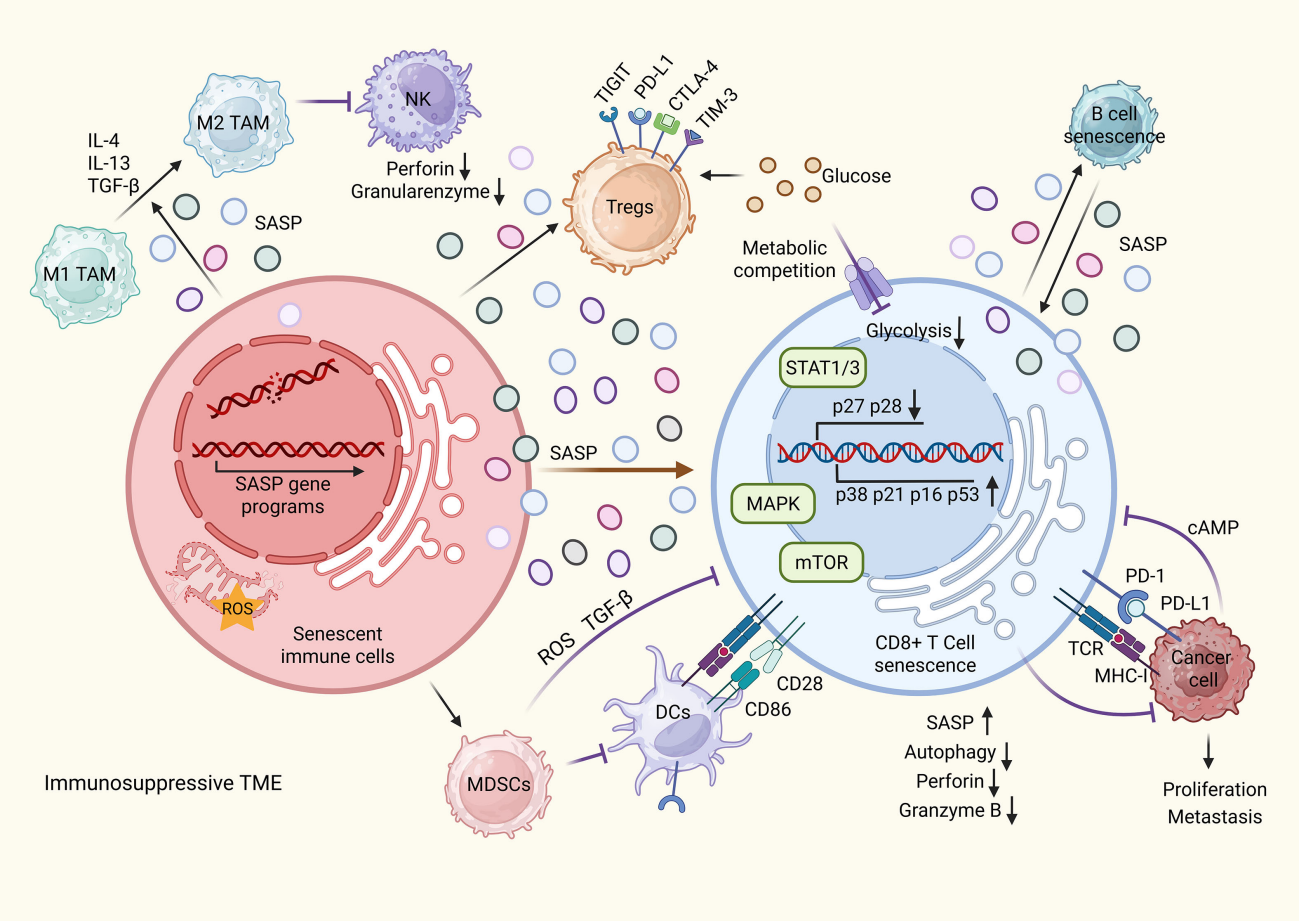
Immune cell senescence drives the formation of an immunosuppressive tumor microenvironment (TME). Senescent immune cells within the TME secrete senescence-associated secretory phenotype (SASP) factors that recruit regulatory T cells (Tregs) and myeloid-derived suppressor cells (MDSCs). These senescent cells can also induce senescence in neighboring immune cells, further amplifying SASP production. SASP factors and immune checkpoint molecules expressed by Tregs and MDSCs activate transcription factors such as mitogen-activated protein kinase (MAPK) and signal transducer and activator of transcription 1/3 (STAT1/3), while concurrently suppressing the mammalian target of rapamycin (mTOR) signaling pathway in CD8^+^ T cells. This dysregulation leads to reduced expression of CD27 and CD28, alongside increased levels of p38, p21, p16, and p53, thereby promoting CD8^+^ T cell senescence. Senescent T cells exhibit impaired autophagy, diminished secretion of perforin and granzyme B, and loss of cytotoxic activity. These effects are exacerbated by elevated cyclic AMP (cAMP) levels resulting from altered tumor metabolism and nutrient competition, especially for glucose, between Tregs and effector T cells. MDSCs further suppress antitumor immunity by inhibiting antigen presentation through recruitment of dendritic cells (DCs). Additionally, SASP components drive the polarization of macrophages from the pro-inflammatory M1 to the immunosuppressive M2 phenotype and impair NK cell-mediated cytotoxicity. Together, these senescence-driven changes facilitate immune evasion, support the development of an immunosuppressive TME, and promote tumor growth and metastasis.

In summary, senescent immune cells significantly reshape the immune landscape within the TME. By fostering an immunosuppressive environment and undermining anti-tumor responses, they contribute to tumor progression and reduced responsiveness to immunotherapy (**Figure 2**). Understanding the mechanisms underlying immune cell senescence and devising strategies to counteract these processes hold promise for enhancing the efficacy of cancer immunotherapy.

### 3.3 T cell senescence and the formation of an immunosuppressive TME

T cell senescence plays a pivotal role in tumor progression through a bidirectional relationship with TME remodeling. While T cell exhaustion and senescence contribute to tumor immune evasion, they are mechanistically distinct. Exhaustion results from chronic antigen exposure and is marked by high expression of inhibitory receptors (e.g., PD-1, CTLA-4), but remains reversible with immunotherapy. In contrast, T cell senescence is driven by cumulative stress and damage, leading to irreversible cell cycle arrest. Importantly, senescent T cells exert paracrine effects that amplify immunosuppression within the TME undermining immunotherapeutic efficacy.

Upon antigen stimulation, naïve T cells undergo TCR rearrangement and metabolic reprogramming involving glucose, glutamine, and fatty acid metabolism. These processes, initiated by antigen-presenting cell (APC) signals and regulated by CD28 the mammalian target of rapamycin complex (mTORC), support activation, proliferation, and differentiation into effector T cells (92, 93). Age-associated CD28 downregulation contributed to reduced generation of CD4^+^/CD8^+^ effector T cells, partially explaining immune deficits in aging and cancer.

Within the TME, Tregs acquire metabolic adaptability through expression of Forkhead box P3 (Foxp3) and sterol O-acyltransferase 2 (SOAT2), which suppress Myc-driven glycolysis and promote oxidative phosphorylation and cholesterol metabolism, facilitating their expansion (94, 95). By competing with effector T cells for glucose, Tregs trigger protein kinase B (AKT)-mediated DNA damage and activate senescence-related pathways (p21, p16, p53, and STAT1/3), leading to T cell senescence (91, 96, 97). Glucose deprivation also induces the mitogen-activated protein kinase (MAPK) cascade, p38 autophosphorylation, further promoting DNA damage and senescence (76). Additionally, γδ Tregs directly induce senescence in naïve, effector T cells and DCs, acquiring potent immunosuppressive capacity (98, 99). While cytotoxic γδ T effectors (encompassing γδT1 and γδT17) can promote tumor progression in response to TME stressors like hypoxia and metabolites, it is unknown if they share the capacity of γδ Treg cells to induce immune cell senescence, a question that warrants further investigation (100, 101). *In vivo* studies show that Tregs can induce senescence in adoptively transferred tumor-specific T cells, thereby impairing the efficacy of CAR-T cells in melanoma models (102). In breast cancer, blockade of PD-L1 and/or STAT3 signaling prevents γδ Treg-induced senescence, enhancing the human epidermal growth factor receptor 2 (HER2)-specific responses and immunotherapy efficacy (99).

TME-derived metabolic byproducts, such as cyclic AMP (cAMP) and adenosine, further exacerbate T cell senescence. Tumor cells transfer intracellular cAMP to effector T cells via gap junctions, inducing DNA damage and accelerating senescence (103). Chronic exposure to extracellular adenosine suppresses telomerase, upregulates caspase-3, and downregulates CD28 by inhibiting its promoter, aggravating T cell aging (104). Senescent T cells also overexpress PD-1, which disrupts TCR signaling by inhibiting zeta-chain-associated protein kinase 70 (ZAP70) phosphorylation and the PI3K–AKT–mTOR axis, while activating p38 signaling (105). These changes impair autophagy and promote the accumulation of dysfunctional, senescent T cells in the TME. Functionally, senescent T cells express reduced levels of perforin and granzyme B, diminishing their cytotoxicity (106–108). In summary, the immunosuppressive TME promotes T cell senescence, which in turn reinforces immune escape and therapeutic resistance. Disrupting this cycle by targeting senescence-associated pathways and restoring effector T cell function represents a promising strategy for improving immunotherapy outcomes.

### 3.4 Senescence of other immune cells and the formation of an immunosuppressive TME

Beyond effector T cells, the senescence of other immune cells—including macrophages, DCs, NK cells, and neutrophils—plays a significant role in shaping an immunosuppressive TME. Macrophages, which may constitute up to 50% of the tumor mass, are particularly susceptible to premature senescence under conditions such as oxidative stress, DNA damage, and chronic inflammation (109). Senescent macrophages suppress T cell-mediated antitumor immunity, reduce the efficacy of ICIs, chemotherapy, and radiotherapy, and are associated with treatment resistance (110, 111). Notably, their experimental depletion enhances tumor control (112). Tumor-resident DCs, especially the CD103^+^ subset, are critical initiators of antitumor immunity and can potentiate responses to PD-L1 blockade while protecting against tumor rechallenge (32). However, Tregs can induce DC senescence through PD-L1 and STAT3 signaling, leading to impaired antigen presentation and diminished immunotherapeutic efficacy (99).

NK cells are key players in innate immunity, mediating tumor surveillance and orchestrating adaptive responses through the release of cytokines, chemokines, and growth factors (25, 113, 114). They recruit and activate DCs, indirectly enhancing T cell-mediated antitumor responses (25), and higher NK cell infiltration correlates with favorable prognosis in various cancer types (115, 116). Yet, in murine models of breast cancer and melanoma, senescent NK cells exhibit impaired cytotoxicity and disrupted glucose and lipid metabolism (117). Neutrophils are also similarly susceptible to TME-induced senescence. In prostate cancer, tumor-secreted apolipoprotein E promotes neutrophil senescence and accumulation of aged-like neutrophils with enhanced immunosuppressive activity (118). Altogether, the accumulation of senescent macrophages, DCs, NK cells, and neutrophils within the TME disrupts antigen presentation, suppresses cytotoxic lymphocyte function, and facilitate immune evasion. These alterations collectively contribute to resistance against multiple forms of cancer therapy, including ICIs, and underscore the need for senescence-targeted strategies to improve immunotherapeutic outcomes.

### 3.5 Dual role of SASP in regulating antitumor immunity

In addition to immune cells and their metabolic products, senescent cells within the TME contribute to tumor progression by transmitting senescence to neighboring cells through autocrine and paracrine mechanisms. SASP factors released by senescent T cells include a wide array of bioactive molecules, such as IL-6, IL-8, monocyte chemoattractant protein-1 (MCP-1), proteases, growth and angiogenic factors, pro-inflammatory cytokines, MMPs, and components of extracellular vesicles (119–121). These molecules reinforce senescence in an autocrine fashion and modulate the behavior of adjacent tumor, stromal, and immune cells via paracrine signaling, thus remodeling the TME (121, 122). In certain contexts, SASP elements—particularly IL-1α, IL-6, and IL-8—promote the recruitment of M1-like macrophages, Th1 cells, and NK cells, facilitating the clearance of senescent tumor cells and suppressing tumor progression (123). These findings highlight that senescence can, under specific conditions, support antitumor immunity. However, persistent accumulation of senescent cells and chronic SASP secretion often lead to the establishment of an immunosuppressive, tumor-promoting environment. For example, aged fibroblasts injected into murine models secrete MMPs that degrade the ECM and release chemokines with pro-angiogenic and pro-tumorigenic activity, promoting tumor cell invasion and proliferation (122, 124). Additionally, SASP-derived IL-6 and IL-8 can drive EMT and enhance MMP expression, further contributing to metastasis (125–127). SASP components such as IL-6 also facilitate the recruitment of MDSCs into the TME. These cells suppress antitumor responses by producing arginase 1, TGF-β, and ROS, thereby inhibiting the activity of CD8^+^ T cells and NK cells (128, 129). Such mechanisms enable cancer cells—including those in breast cancer—to evade immune surveillance and expand despite immune pressure. In gastric cancer, TNF-α promotes the expansion of CD45RA^−^CCR7^−^ Treg subsets, which suppress CD8^+^ T cell function via STAT3 signaling (130). Senescent cell-derived exosomes also act as SASP vectors, carrying oncogenic cargo—including IL-6, mesenchymal–epithelial transition factor (MET), and pro-metastatic miRNAs—that activate PI3K/STAT3 and Wingless/Integrated (Wnt) signaling to drive proliferation, EMT, and angiogenesis. These vesicles may also deliver immunosuppressive molecules such as TGF-β, PD-L1, and ligands for natural killer group 2 member D, suppressing DC maturation and NK/CD8^+^ T cell cytotoxicity. Moreover, they can transfer oncogenic factors like human telomerase reverse transcriptase (hTERT) mRNA, ΔNp73, and cytoplasmic DNA, which promote chromosomal instability and chemoresistance (131, 132). This exosome-mediated communication further establishes a secondary SASP response in recipient cells, sustaining an inflammatory, immune-suppressive, and tumor-supportive niche. Additionally, senescent B cells expressing p16^Ink4a^ contribute to poor immunotherapy responses, as observed in bladder cancer. These cells activate p38/MAPK signaling and secrete SASP factors that further suppress antitumor immunity (133, 134). Overall, the influence of SASP on tumor immunity is highly context-dependent. While transient SASP activity in early tumorigenesis may enhance immunosurveillance and tumor suppression, persistent SASP signaling in advanced disease promotes tumor progression and immune evasion (135). Therefore, a nuanced understanding of the spatiotemporal dynamics of SASP is essential. Therapeutic strategies that selectively attenuate the chronic, pro-tumorigenic aspects of SASP while preserving its acute antitumor effects could significantly enhance the efficacy of immunotherapies.

## 4 The limitations of immune cell senescence on immunotherapy applications

Cancer immunotherapy, which aims to harness the immune system to eliminate tumors and prevent relapse, has revolutionized clinical cancer treatment. Major strategies include ICIs, cell-based immunotherapies, cancer vaccines, and immune modulators (**Table 1**). These approaches share a common goal: to remodel the TME and enhance effector T cell function. However, the accumulation of senescent immune cells within the TME poses a significant barrier to immunotherapy efficacy. These senescent cells contribute to immune dysfunction, reduce cytotoxic responses, and propagate immunosuppressive signaling, thereby undermining therapeutic outcomes. Consequently, a deeper understanding of immune cell senescence within the TME is critical to overcoming resistance and optimizing the benefits of immunotherapy. Integrating senolytic agents or anti-senescence strategies with existing immunotherapies may provide a promising avenue to eliminate dysfunctional immune cells or restore their function. Such combinatorial approaches could help overcome immunotherapy resistance and enhance the efficacy and durability of treatment responses.

**Table 1:** Overview of immunotherapy drugs: clinical use and ongoing trials.

Name	Clinical stage	Applicable tumors	Mechanism of action	Current limitations	References
PD-1/PD-L1 inhibitors					
Pembrolizumab	FDA-approved	NSCLC, Melanoma, etc.	Blocks PD-1/PD-L1 pathway, restoring T cell cytotoxic function	Poor response in low-mutational-load tumors	(237–239)
Nivolumab	FDA-approved	HCC, NSCLC, etc.	Blocks PD-1/PD-L1 pathway	irAEs; requires long-term monitoring	(237–239)
Atezolizumab	FDA-approved	TNBC, Urothelial Carcinoma	Blocks PD-1/PD-L1 pathway	May cause fatigue, rash	(237–239)
CA-170	Phase II Clinical Trial	Multiple solid tumors	Dual inhibition of PD-L1 and VISTA immune checkpoints	irAEs	(240)
CTLA-4 inhibitors					
Ipilimumab	FDA-approved	Metastatic Melanoma	Blocks CTLA-4, enhancing T cell activation	Diarrhea/colitis	(241)
Tremelimumab	Phase III Clinical Trial	HCC, Biliary Tract Cancer	Combined targeting of PD-L1 and CTLA-4, activating T cells	Limited efficacy as monotherapy	(242)
LAG-3 inhibitors					
Relatlimab	FDA-approved	Melanoma (combined with nivolumab)	Blocks LAG-3, enhancing T cell infiltration	Fatigue, rash	(243)
TIGIT Inhibitors					
Tiragolumab	Phase III Clinical Trial	NSCLC,SCLC	Blocks TIGIT-CD155 interaction, reversing T/NK cell inhibition; combines with PD-L1 inhibitors	Efficacy dependent on PD-L1 expression; mixed clinical outcomes	(244, 245)
Vibostolimab	Phase II/III Clinical Trial	NSCLC, Melanoma	Blocks TIGIT-CD155 pathway	Efficacy linked to PD-L1 status; common adverse events: pruritus, hypoalbuminemia	(244, 245)
TIM-3 inhibitors					
Sabatolimab	Phase III Clinical Trial	MDS, AML	Blocks TIM-3 binding to ligands, reversing T cell exhaustion; combinable with PD-1 inhibitors	Hematologic toxicity; limited monotherapy efficacy	(246)
Sym023	Phase I/II Clinical Trial	Advanced Solid Tumors, Lymphoma	Blocks TIM-3 pathway	Fatigue, infusion reactions	(247)
ACT therapies					
CAR-T Cell Therapy	FDA-approved	Large B-cell Lymphoma, Leukemia	Genetically engineered T cells targeting tumor antigens	Limited efficacy in solid tumors; off-target toxicity; CRS	(248)
Lifileucel	FDA-approved	Advanced Melanoma, Cervical Cancer	Expands TILs for reinfusion	Complex logistics; high toxicity	(249–251)
Ad-RTS-hIL-12	Phase I/II Clinical Trial	Melanoma, Brain Tumors	TIL-mediated IFN-γ production	High toxicity; risk of genomic integration	(252)
Other therapeutic agents					
T-VEC	FDA-approved	Melanoma	Lyses tumor cells, activating APCs	Limited applicability	(248)
Sipuleucel-T	FDA-approved	Prostate Cancer	Delivers tumor antigens to activate T cells	Complex manufacturing; high cost; restricted usage	(248)
Tebentafusp	FDA-approved	Melanoma	Engineered TCR targeting gp100 antigen, activating T cells	High cost; narrow applicability	(251)
KN046	Phase II Clinical Trial	HCC, NSCLC	Simultaneously blocks PD-L1 and CTLA-4, enhancing synergistic effects	Dual targeting may increase risk of irAEs	(253)
Oleclumab	Phase I/II Clinical Trial	TNBC	Inhibits CD73-mediated adenosine production, alleviating immunosuppressive microenvironment	Fatigue, diarrhea	(254)

PD-1, Programmed cell death protein 1; PD-L1, Programmed death-ligand 1; CTLA-4, Cytotoxic T-lymphocyte-associated protein 4; LAG-3, Lymphocyte-activation gene 3; TIGIT, T-Cell immunoreceptor with Ig and ITIM domains; TIM-3, T Cell immunoglobulin and mucin-domain containing-3; VISTA, V-domain Ig suppressor of T cell activation; CD73, Cluster of differentiation 73; ACT, Adoptive cell transfer; CAR-T, Chimeric antigen receptor T cell therapy; TIL, Tumor-Infiltrating lymphocytes; TCR, T cell receptor; APCs, Antigen-presenting cells; Treg, Regulatory T Cells; NK, Natural killer; IFN-γ, Interferon gamma; CRS, Cytokine release syndrome; NSCLC, Non-small cell lung cancer; HCC, Hepatocellular carcinoma; TNBC, Triple-negative breast cancer; SCLC, Small cell lung cancer; AML, Acute myeloid leukemia; MDS, Myelodysplastic syndromes; irAEs, Immune-related adverse events; gp100, Glycoprotein 100; FDA, Food and Drug Administration.

### 4.1 Immune checkpoint inhibitors

Immune checkpoints are inhibitory receptors on T cells exploited by the TME to suppress anti-tumor immunity and promote exhaustion. Immune checkpoint inhibitors (ICIs) block these pathways, restoring T cell function and revolutionizing cancer treatment, with efficacy in melanoma, NSCLC, renal cell carcinoma, and triple-negative breast cancer, though resistance remains a challenge (136–139). Key checkpoints include PD-1, which attenuates T cell signaling upon binding PD-L1, and CTLA-4, which inhibits T cell activation by competing with CD28 (140, 141). Clinically approved ICIs—such as pembrolizumab, nivolumab, and ipilimumab —have demonstrated significant benefit in multiple cancer types. To overcome resistance, combination therapies—such as dual checkpoint blockade (e.g., PD-1 plus CTLA-4) or pairing ICIs with chemotherapy, radiotherapy, or targeted agents—are being actively tested. Novel checkpoints like lymphocyte activation gene-3 (LAG-3), T cell immunoreceptor with immunoglobulin and immunoreceptor tyrosine-based inhibitory motif domains (TIGIT), and T cell immunoglobulin and mucin domain-containing protein 3 (TIM-3) also play non-redundant roles in immunosuppression; while monotherapies against them have limited efficacy, combinations with PD-1 blockade show enhanced anti-tumor responses (142–151). For example, the LAG-3 inhibitor relatlimab combined with nivolumab is U.S. Food and Drug Administration (FDA)-approved for melanoma, while TIGIT and TIM-3 inhibitors are primarily being evaluated in combination with PD-1 inhibitors (152–154). Ongoing studies are exploring dual or multi-modal regimens to overcome resistance in solid tumors and expand the proportion of patients who benefit from immunotherapy.

### 4.2 Adoptive cell therapy

In recent years, ACT has emerged as a promising immunotherapeutic strategy to overcome T cell exhaustion within TME, encompassing approaches such as tumor-infiltrating lymphocytes (TILs), CAR-T cells, and TCR-engineered T cells. TIL therapy involves expanding and reinfusing a patient’s naturally tumor-reactive T cells, showing strong efficacy in melanoma but limited by complex manufacturing (155–157). CAR-T cells are genetically modified to target surface antigens independently of MHC, achieving remarkable success in hematologic malignancies but struggling in solid tumors due to poor infiltration and immunosuppression; next-generation constructs and alternative platforms like CAR-natural killer (CAR-NK) and CAR-macrophage (CAR-M) are being explored to enhance efficacy (158–162). TCR-T therapy, which engineers T cells to target MHC-presented intracellular antigens, offers deeper tumor penetration but is constrained by human leukocyte antigen (HLA) matching, off-target toxicity risks, and limited antigen availability (163–166). While ACT has shown substantial success in hematologic malignancies, its efficacy in solid tumors remains limited—largely due to immune aging and the immunosuppressive nature of the TME (167, 168). Therefore, future advances will likely rely on combinatorial strategies—such as improving cell persistence, developing novel constructs to resist the TME, and combining ACT with immune checkpoint blockade—to achieve broader and more durable anti-tumor responses.

### 4.3 Cancer vaccines

Cancer vaccines aim to activate the immune system to generate antigen-specific T cells that mediate tumor regression (169). Current efforts focus on neoantigen-based vaccines, which arise from somatic mutations and are uniquely expressed by tumor cells (170, 171). Unlike tumor-associated antigens, neoantigens are recognized as non-self, avoiding central tolerance and enabling the induction of high-affinity cytotoxic T lymphocytes with minimal off-target toxicity (172). This makes them ideal immunotherapy targets due to their high immunogenicity and tumor specificity. Personalized cancer vaccines (PCVs) are developed by sequencing tumor and matched normal samples to identify patient-specific neoantigens, which are then formulated into mRNA- or peptide-based platforms. These vaccines elicit targeted immune responses that selectively eliminate tumor cells while sparing normal tissues (173). Clinical trials have demonstrated that PCVs can reduce recurrence and prolong disease-free survival in high-risk patients, including those with renal cell carcinoma, with durable vaccine-specific T cell responses persisting over 36 months (174). The clinical efficacy of ICIs correlates with neoantigen burden (175, 176), supporting the combination of PCVs with ICIs to enhance outcomes. Despite progress, the efficacy of neoantigen vaccines (e.g., LK101 injection) is limited by the immunosuppressive TME (177–179). Factors such as Tregs, MDSCs, and immunosuppressive cytokines, along with vaccine-induced resistance, diminish PCV immunogenicity (180). Overcoming these challenges requires deeper understanding of the TME and strategies to modulate its immunosuppressive properties. Optimizing PCV design and clinical utility will depend on rational combination therapies, improved antigen selection, and real-time immune monitoring.

### 4.4 Immunotherapy resistance and immune cell senescence

Although immunotherapy has improved treatment response and safety relative to conventional chemotherapy, many cancers eventually develop resistance (11). This resistance is multifactorial, involving tumor-intrinsic and immune-related mechanisms. Among these, immune cell senescence and the resulting immunosuppressive TME contribute substantially to therapeutic failure. For instance, loss of HLA class I—a key mediator of antigen presentation—through genetic mutations or epigenetic silencing impairs tumor immune recognition (181, 182).

Clinical studies have shown that while PD-1 inhibitors improve survival in younger (<65 years) and older (≥65 years) patients, no survival benefit is observed in those aged ≥75 years compared to chemotherapy (183). Similarly, ICIs show limited efficacy in reversing T cell exhaustion in glioblastoma multiforme (GBM) (184). In NSCLC, elevated levels of circulating CD57^+^KLRG-1^+^ CD8^+^ T have been associated with poor ICI responses (185). Aged mouse models of melanoma have demonstrated reduced anti–PD-L1 efficacy due to impaired DC migration and diminished CD8^+^ T cell responses—effects that were partially reversed by DC activators (186). Likewise, triple-negative breast cancer models have shown deficient CD8^+^ T cell activation in aged mice, rendering ICIs ineffective (187). Similar patterns of age-related ICI resistance have been reported in colon cancer and lymphoma models (74). Senescent Tregs and MDSCs accumulate in the TME and suppress CD8^+^ T cells via IL-10, TGF-β, PD-1/PD-L1, and TIGIT/CD155 signaling pathways, further contributing to anti–PD-L1 therapy resistance. Moreover, metabolic dysfunction in senescent immune cells leads to upregulation of IDO activity, resulting in L-tryptophan depletion and accumulation of immunosuppressive metabolites such as N-formylkynurenine, which further attenuate antitumor immunity (188, 189).

Peripheral senescent T cells—often induced by chronic antigen exposure or extensive chemotherapy—can impair the function of adoptively transferred cells and reduce the efficacy of cancer vaccines. In melanoma models, senescent immune cells have been shown to diminish CAR-T cell efficacy (102). Preconditioning T cells to mitigate senescence-associated phenotypes can improve the specificity and durability of ACT therapies. For instance, engineering CAR-T cells with dual co-stimulatory domains (CD28 and 4-1BB) enhances metabolic fitness and resistance to senescence, resulting in improved antitumor responses (190). Co-treatment with CAR-NK and CAR-T cells in multiple myeloma has been shown to restore co-stimulatory molecule expression and delay T cell senescence (191). Similarly, early Phase III trial data for CimaVax-EGF, a therapeutic vaccine for stage IIIB/IV NSCLC, suggest reduced efficacy in the context of T cell senescence (192). Furthermore, the effectiveness of neoantigen vaccines depends on a stable and diverse TCR repertoire, which is often compromised in aged or senescent immune systems. In summary, immune cell senescence promotes the development of a suppressive TME and contributes to immunotherapy resistance through multiple mechanisms.

Cancer stem cells (CSCs) are now recognized as primary drivers of therapeutic resistance to immunotherapy (193). By downregulating MHC class I, NKG2D ligands, and neoantigens, CSCs reduce their immunogenicity. Concurrently, they secrete immunosuppressive cytokines such as TGF-β, IL-6, and CCL2/CCL5 to recruit M2-polarized TAMs, MDSCs, and Tregs, while upregulating “don’t-eat-me” signals—including PD-L1, CD47, and CD155—to inhibit cytotoxic T cell, NK cell, and macrophage responses (194, 195). Their intrinsic plasticity allows CSCs to enter slow-cycling or EMT-mediated dormancy states, enabling rapid phenotypic switching under immune pressure. This adaptability constructs a highly immunosuppressive, low-immunogenic, and cytotoxic escape network. Although direct evidence linking CSCs to immune cell senescence remains limited, the functional overlap between CSC-mediated immune evasion and senescence-associated immune dysfunction suggests potential crosstalk between CSCs and senescent immune cells. Together, they may synergistically shape an immunosuppressive TME and contribute to resistance against immune-based therapies. Addressing senescence-associated immune dysfunction may therefore be critical to overcoming resistance and enhancing the long-term efficacy of cancer immunotherapy.

### 4.5 Special considerations for immunotherapy in elderly cancer patients

Aging induces structural and functional changes in immune organs such as the thymus, bone marrow, spleen, and lymph nodes, resulting in impaired immune surveillance, reduced antigen presentation, accumulation of immunosuppressive Tregs and memory lymphocytes, and diminished cytotoxic CD8^+^ T cell responses (196–199). These age-associated immune alterations significantly limit the efficacy of immunotherapy in elderly patients. Although some studies report elevated PD-L1 expression in older individuals, its predictive value for immunotherapy response remains inconclusive (200, 201). Compared to younger patients, the elderly exhibit more pronounced immune cell senescence, contributing to tissue degeneration, comorbidities, and a systemic pro-inflammatory state driven by SASP. This chronic inflammation exacerbates T cell exhaustion and senescence, increases tumor susceptibility, and dampens immunotherapeutic responses (202). For example, Huff et al. reported increased levels of senescent T cells in the TME and peripheral blood of patients with GBM, indicating systemic impairment of immune responses (184). Age-related changes in the TME also hinder immunotherapy efficacy. In aged mice, altered immune cell composition—particularly enhanced emergency myelopoiesis—accelerates lung tumor progression. IL-1α is upregulated in lung tumors of older mice, promoting myelopoiesis and immunosuppression. Notably, blockade of IL-1α signaling delayed tumor growth and enhanced NK cell-mediated immunity (203).

ICIs, while beneficial, are associated with immune-related adverse events (irAEs), which can cause multi-organ toxicities and reduce treatment tolerance. These events are thought to result from reactivation of autoreactive T cells. Advanced age is a known risk factor for irAEs, with frail older adults experiencing higher rates of hospitalization, longer hospital stays, and more frequent ICI discontinuation (204). Despite the increasing use of ICIs in clinical practice, elderly individuals remain underrepresented in clinical trials, limiting available safety and efficacy data for this population. Therefore, further research is essential to determine the risk–benefit ratio of immunotherapy in older adults.

## 5 Combined therapeutic strategies: senolytics and immunotherapy

Cellular senescence serves as a double-edged sword in cancer biology. In the precancerous phase, clearing senescent cells using immunotherapy or senotherapeutics can prevent tumor initiation—particularly beneficial in the elderly, where senescent cell accumulation and SASP contribute to a pro-tumorigenic microenvironment. However, in established tumors, anti-cancer therapies and metabolic stress can induce detrimental senescence in immune cells, fostering an immunosuppressive TME that undermines the effectiveness of immunotherapy. Strategically timed combinations of senotherapeutics and immunotherapy offer promising avenues to restore anti-tumor immunity and enhance therapeutic outcomes. However, the timing of senescence-targeting intervention is critical: interventions introduced too early may disrupt therapy-induced tumor suppression, while delayed application may allow irreversible SASP-mediated damage to accumulate. This highlights the urgent need for precise biomarkers that can guide the optimal timing of intervention. Additionally, developing targeted senotherapeutics capable of selectively eliminating harmful senescent cells—while sparing beneficial ones such as functionally recovering immune cells or quiescent stem cells—remains a vital complementary strategy (**Table 2**).

**Table 2:** Anti-senescence agents used in combination with immunotherapy.

Drugs	Therapeutic target	Clinical/preclinical applications	Combination product	NCT number or References
Metformin	mTOR and Complex I	Cervical, Vaginal, and Vulvar Cancers; Phase II	HPV Vaccine and Imiquimod	NCT06686043
		Refractory Microsatellite Stable Metastatic Colorectal Cancer; Phase II	Nivolumab	NCT03800602
		Stage III-IV Non-small Cell Lung Cancer That Cannot Be Removed by Surgery; Phase II	Nivolumab	NCT03048500
		Small Cell Lung Cancer; Phase II	PD-1 inhibitor (Sintilimab)	NCT03994744
		Metastatic Breast Cancer and Triple-negative Breast Cancer; Phase II	EGCG, Quercetin, and zinc	NCT05680662
Quercetin	Oxidative Stress	Metastatic Breast Cancer and Triple-negative Breast Cancer Phase II	Epigallocatechin gallate, Metformin, and zinc	NCT05680662
Dasatinib		Metastatic Melanoma; Phase II	Dendritic Cell Vaccines	NCT01876212
		B-cell Acute Lymphoblastic Leukemia; Phase III	Chemotherapy and Blinatumomab	NCT06124157
Rapamycin	mTOR	NY-ESO-1 Expressing Solid Tumors; Phase I	Vaccine Therapy	NCT01522820
		Relapsed and Refractory Multiple Myeloma; Phase Ib	Pomalidomide, and Dexamethasone	NCT03657420
		PD-(L)1 Resistant Solid Tumors; Phase I/II	Autologous Rapamycin-Resistant Th1/Tc1 Cell Therapy	NCT05144698
Everolimus		Colorectal Cancer; Phase	PD-1	NCT06301386
Temsirolimus		Advanced or Metastatic Malignancy; Phase I	Bevacizumab and Valproic Acid, or Cetuximab	NCT01552434
Ruxolitinib Phosphate	JAK2	Metastatic Stage IV Triple Negative Breast Cancer; Phase I	Pembrolizumab	NCT03012230
VTX-2337	TLR 8	Squamous Cell Carcinomas of the Head and Neck; Phase I	Cetuximab	NCT01334177
β-Glucan	MDSC	Lung Cancer; Phase II	Vaccine 1650-G	NCT01829373
ABT-263	BCL-2	Preclinical	-	(209–212)
ABT-737				
Resveratrol	NRF2	Preclinical	-	(215–218)
Digoxin	Na+/K+ pumps	Preclinical	-	(209–212)
Ouabain				
Cetuximab	IL-6	Preclinical	-	(220, 221)

The NCT numbers in the table are from the ClinicalTrials website. mTOR, Mammalian target of rapamycin; HPV, Human papillomavirus; PD-1, Programmed cell death protein 1; EGCG, Epigallocatechin gallate; NY-ESO-1, New York Esophageal Squamous Cell Carcinoma 1; Th1, Helper T cell 1; Tc1, Cytotoxic T cell 1; JAK2: Janus kinase 2; Toll-like receptor 8; MDSC, Myeloid-derived suppressor cell; Bcl-2, B-cell lymphoma-2; NRF2, Nuclear factor erythroid 2-related factor 2; IL-6, Interleukin-6.

### 5.1 Potential of anti-aging drugs as adjuvants in antitumor therapy

Metformin, a traditional antidiabetic agent, has emerged as a promising candidate with anti-aging and antitumor properties (205). It inhibits mitochondrial complex I and mTOR signaling, thereby lowering systemic glucose utilization, enhancing CD8^+^ T cell-mediated tumor clearance, and improving the efficacy of anti-PD-1 immune checkpoint blockade (206). By suppressing glycolytic metabolism, metformin also facilitates the development of memory T cells, further strengthening long-term antitumor immunity (207). Multi-omics analyses of Treg metabolism suggest that targeting shared metabolic pathways between Tregs and tumor cells can disrupt Treg homeostasis and phenotypic stability. This strategy offers a selective and controllable approach for depleting Tregs within the TME (208).

First-generation senolytic agents—including B-cell lymphoma-2 (Bcl-2) family inhibitors (ABT-263, ABT-737), dasatinib and quercetin combinations, and cardenolides (e.g., ouabain, digoxin)—have been successfully employed alongside radiotherapy and chemotherapy to eliminate therapy-induced senescent cells, thereby limiting tumor progression and metastasis (209–212). For example, Maggiorani et al. (74) demonstrated that combining the Bcl-2 inhibitor ABT-263 with immunotherapy enhances therapeutic efficacy by clearing senescent cells and restoring immune homeostasis within the TME, ultimately improving survival outcomes. Emerging senolytic strategies include engineered CAR-T cells targeting senescent cell-specific surface markers. Urokinase-type plasminogen activator receptor (uPAR), commonly upregulated on senescent cells, has been exploited for uPAR-specific CAR-T cell therapies to selectively eliminate senescent cells *in vitro* and *in vivo*, leading to improved outcomes in mouse models of lung cancer and liver fibrosis (213). Additionally, recent work has identified bifunctional apoptosis regulator (BFAR) as a critical modulator enriched in senescent CD8^+^ T cells. BFAR restricts STAT1-mediated reprogramming of tissue-resident memory T cells by regulating JAK2 deubiquitination. Inhibition of BFAR using the small molecule iBFAR2 restores memory T cell generation and rescues antitumor activity in senescent or anti-PD-1-resistant CD8^+^ T cells (58). Collectively, senolytic agents offer a compelling strategy to enhance cancer immunotherapy by clearing senescent cells, reactivating immune effector functions, and mitigating the immunosuppressive effects of the TME. However, their application requires careful consideration of tumor type, therapeutic timing, and the patient’s immune status to avoid off-target effects and optimize therapeutic benefit.

### 5.2 Development and challenges of SASP inhibitors

Given the critical role of SASP in promoting tumor progression and therapy resistance, targeting SASP presents a promising alternative to senolytics. This approach aims to mitigate the deleterious effects of persistent senescent cells while preserving their transient benefits in immunosurveillance (214). Key regulators of the SASP include the mTOR and NF-κB pathways. Studies have shown that metformin (which inhibits NF-κB nuclear translocation), rapamycin (an mTOR inhibitor), and resveratrol (an activator of the nuclear factor erythroid 2-related factor 2 [Nrf2] pathway) can suppress SASP expression, exerting anti-aging and anti-tumor effects (215–218). Notably, metformin has also been shown to enhance the efficacy of ICIs, highlighting its potential for cancer immunoprevention and treatment (219). Additionally, monoclonal antibodies targeting specific SASP factors have been explored. For example, cetuximab, an anti-IL-6 antibody, has been used to treat multicentric Castleman’s disease and is currently under investigation in various cancer types (220, 221).

Despite these advances, the indiscriminate inhibition of SASP poses significant challenges. Cytokines such as IL-6 and IL-8 are essential for normal immune responses; their sustained suppression may compromise immune activation, increase susceptibility to infections, and disrupt immune homeostasis. Paradoxically, this could lead to chronic inflammation, immune tolerance, and impaired anti-tumor immunity. Moreover, the heterogeneity of the SASP complicates its clinical translation. SASP composition varies depending on tissue type, cellular origin, the nature of the senescence-inducing stimulus, and temporal context. For example, different senescence inducers in hepatocellular carcinoma elicit distinct SASP profiles: CX5461 predominantly induces the IL-8/CXCL10 axis, while alisertib upregulates VEGF (222). Even within the same tumor type, SASP signatures may differ due to genetic background, cell lineage, or donor variability (223). Therefore, the effective development of SASP inhibitors will require: selective targeting of tumor-promoting SASP components while preserving immune-activating elements; precision molecular strategies that integrate immune monitoring and patient-specific profiling; and combinatorial approaches with immunotherapies to maximize efficacy and minimize immunosuppression.

Ultimately, refining SASP modulation will be essential for translating this promising approach into safe and effective personalized cancer therapies.

### 5.3 Prospects of nanodelivery systems in combination therapy

Conventional immunotherapies and senotherapeutics are often hindered by systemic toxicity, poor pharmacokinetics, and limited specificity (224). Nanodelivery systems offer a promising solution by enhancing drug targeting, stability, and bioavailability. Surface modifications with antibodies, peptides, or ligands—such as folic acid or HER2—enable active targeting of tumor cells (225). Nanoparticles (10–100 nm) also exploit the enhanced permeability and retention effect of tumor vasculature for passive targeting. Additionally, nanocarriers protect therapeutic agents from enzymatic degradation, extend circulation time, and concentrate drugs at tumor sites, thereby minimizing off-target effects.

A variety of nanomaterials have been developed to enhance anti-aging and anticancer therapies. For example, mesoporous polydopamine nanoparticles coated with galactan and loaded with dasatinib and quercetin can respond to high β-galactosidase activity and acidic pH, effectively clearing chemotherapy-induced senescent cells and suppressing breast cancer progression and metastasis (226). Similarly, mPEG-PLGA-PLL nanoparticles (composed of methoxy polyethylene glycol, poly[lactic-co-glycolic acid], and poly-L-lysine) conjugated with PD-L1-blocking antibodies enhance early CD8^+^ T cell immunosurveillance, reverse T cell dysfunction, and prevent immune escape (227). Nanostructures incorporating tumor antigens have demonstrated therapeutic promise in preclinical models of melanoma, thymoma, and lymphomas. Once administered, these structures are preferentially internalized by DCs, which stimulates robust effector T cell and antibody responses. ultimately improving survival outcomes (228, 229). In cancer vaccine formulations, glycosylated PLGA nanoparticles have been employed to co-deliver ovalbumin and CpG oligonucleotides as adjuvants, enhancing immune activation (230). Another innovative approach involves magnetic nanoparticles (Fe_3_O_4_) loaded with sulfamethazine and cloaked with platelet membranes (Fe_3_O_4_-SAS@PLT). These induce ferroptosis in tumor cells while reprogramming M2-like macrophages into M1-like phenotypes, thereby disrupting the immunosuppressive TME and enhancing ICI efficacy (231). The integration of nanotechnology, cellular senescence modulation, and immunotherapy represents a cutting-edge direction for developing safer, more precise, and highly effective cancer treatments. This convergence holds significant potential for advancing personalized immunotherapy and overcoming current limitations in cancer therapy.

## 6 Conclusion and perspectives

The self-perpetuating feedback loop between senescent immune cells and the immunosuppressive TME represents a major obstacle to effective cancer immunotherapy. Senescent immune cells not only lose their intrinsic antitumor functions but also secrete SASP components—including IL-6, CXCLs, and IL-10—that recruit and activate immunosuppressive populations such as Tregs and MDSCs, while promoting macrophage polarization toward the M2 phenotype. These effects collectively intensify immunosuppression within the TME. Conversely, TME-associated stressors such as hypoxia, metabolic reprogramming, and upregulated immune checkpoint signaling (e.g., PD-1/PD-L1) accelerate immune cell senescence. This bidirectional crosstalk establishes a complex regulatory network that facilitates immune evasion and drives resistance to immunotherapy. Targeting immune senescence and SASP-related signaling pathways presents a compelling strategy for reversing therapeutic resistance. As highlighted in this review, a diverse array of agents—including metformin, ABT-263, other senolytics, SASP inhibitors, and nanotechnology-based drug delivery platforms—have demonstrated potential in preclinical models to eliminate senescent cells, restore immune competence, and reprogram the TME. Among these, nanodelivery systems offer unique advantages in drug stability, specificity, and controlled release, expanding the landscape of combination strategies. The integration of senescence-targeted therapeutics with established immunotherapies—such as ICIs and CAR-T cells—represents a promising direction for next-generation precision oncology.

Despite considerable progress in elucidating the interplay between immune senescence and the TME, a major challenge remains: the absence of robust, specific, and sensitive biomarkers to accurately monitor immune senescence and immune suppression within the TME. Emerging high-dimensional technologies such as single-cell RNA sequencing, multiplex imaging, and spatial profiling provide exciting opportunities to address this gap. Advanced spatially resolved tools—including tissue-based cyclic immunofluorescence, imaging mass cytometry, and CODEX—enable detailed analysis of the spatial distribution and heterogeneity of senescent immune cells across different tumor types and patient populations (232–234). These platforms are instrumental in characterizing cellular interactions within the TME and guiding precision immunotherapeutic interventions. Notably, recent single-cell transcriptomic studies incorporating TCR sequencing have identified Granzyme K^+^ CD8^+^ T cells as a conserved marker of inflammatory senescence, whose prevalence increases with age (235, 236). Integrative multi-omics approaches combining flow cytometry, single-cell transcriptomics, and epigenetic clocks offer powerful means to identify and validate novel biomarkers. These biomarkers are critical for predicting immunotherapy response, selecting patients likely to benefit, and enabling real-time monitoring of treatment efficacy.

In summary, this evolving understanding of immune cell senescence and its reciprocal interaction with the TME paves the way for personalized, precision-guided cancer immunotherapy. By assessing immune senescence status, TME characteristics, and tumor mutational burden, clinicians can refine patient stratification and optimize the timing and composition of immunotherapeutic regimens. Looking ahead, the incorporation of validated biomarkers, widespread application of single-cell technologies, artificial intelligence–driven predictive modeling, and innovations in senolytic and nanomedicine platforms hold the potential to elevate the efficacy, safety, and personalization of cancer immunotherapy. These advances offer new hope for improving clinical outcomes and quality of life for patients facing cancer.

## Glossary

ACT: Adoptive Cell Therapy
AKT: Protein kinase B
APCs: Antigen-presenting cells
Bcl-2: B-cell lymphoma-2
BFAR: Bifunctional apoptosis regulator
Btk: Bruton’s tyrosine kinase
cAMP: Cyclic AMP
CAFs: Cancer-associated fibroblasts
CAR: Chimeric antigen receptor
CAR-T: Chimeric antigen receptor T cell
CCL: Chemokine (C-C motif) ligand
CCR5: C-C chemokine receptor type 5
CEACAM1: Cell adhesion molecule 1
cDCs: Classical dendritic cells
CODEX: CO-Detection by indEXing
CSC: Cancer stem cell
CTLA-4: Cytotoxic T lymphocyte-associated protein 4
CXCL6: C-X-C motif chemokine ligand 6
DCs: Dendritic cells
ECM: Extracellular matrix
EGFR: Epidermal growth factor receptor
EGFR-19 del: EGFR exon 19 deletion
EMT: Epithelial-mesenchymal transition
ER: Endoplasmic reticulum
GAC: Gastric adenocarcinoma
GBM: Glioblastoma multiforme
HER2: Human epidermal growth factor receptor 2
HLA: Human leukocyte antigen
HMGB1: High mobility group box 1
ICOS: Inducible T cell co-stimulator
ICIs: Immune checkpoint inhibitors
IDO: Indoleamine 2,3-dioxygenase
IFN: Interferon
IL: Interleukin
iNOS: Inducible nitric oxide synthase
irAEs: Immune-related adverse events
ITIM: Tyrosine-based inhibition motif
JAK2: Janus kinase 2
KLRG-1: Killer cell lectin-like receptor G1
LAG-3: Lymphocyte Activation Gene-3
MAPK: Mitogen-activated protein kinase
MCP-1: Monocyte chemoattractant protein-1
MDSCs: Myeloid-derived suppressor cells
MHC: Major histocompatibility complex
MMPs: Matrix metalloproteinases
mTORC: Mammalian target of rapamycin complex
NK: Natural killer
NKRs: Natural killer receptors
NSCLC: Non-small cell lung cancer
PD-1: Programmed cell death protein 1
PD-L1: Programmed death-ligand 1
PI3K: Phosphatidylinositol-3-kinase
PLGA: Poly(lactic-co-glycolic acid)
PLL: Poly-L-lysine
PtdSer: Phosphatidylserine
REP: Rapid expansion protocol
ROS: Reactive oxygen species
SASP: Senescence-associated secretory phenotype
SH2: Src homology 2
SOAT2: Sterol O-acyltransferase 2
STAT1/3: Signal transducer and activator of transcription 1/3
TCR: T cell receptor
TCR-T: T cell receptor–engineered T cell
TEXs: Exhausted T cells
TIGIT: T cell immunoreceptor with immunoglobulin and immunoreceptor tyrosine-based inhibitory motif domains
TIL: Tumor-infiltrating lymphocytes
TIM-3: T cell immunoglobulin and mucin domain-3
TME: Tumor microenvironment
Tregs: Regulatory T cells
uPAR: Urokinase-type plasminogen activator receptor
ZAP-70: Zeta-chain-associated protein kinase 70.

## References

[1] Bray F Laversanne M Sung H Ferlay J Siegel RL Soerjomataram I. Global cancer statistics 2022: GLOBOCAN estimates of incidence and mortality worldwide for 36 cancers in 185 countries. CA Cancer J Clin. (2024) 74:229–63. doi: 10.3322/caac.21834, PMID: 38572751

[2] Möller K Löwe A Jenssen C Bhutani MS On W Everett SM. Comments and illustrations of the European Federation of Societies for Ultrasound in Medicine contrast-enhanced ultrasound guidelines. Rare pancreatic tumors, imaging features on transabdominal ultrasound and EUS with contrast enhancement: Rare epithelial pancreatic tumors: solid pseudopapillary neoplasm, acinar cell carcinoma, mixed neuroendocrine-non-neuroendocrine neoplasms, some rare subtypes of pancreatic adenocarcinoma and pancreatoblastoma. Endosc Ultrasound. (2024) 13:129–44. doi: 10.1097/eus.0000000000000056, PMID: 39318646 PMC11419495

[3] Mahajan S Siyu S Bhutani MS. What can artificial intelligence do for EUS? Endosc Ultrasound. (2025) 14:1–3. doi: 10.1097/eus.0000000000000102, PMID: 40151598 PMC11939944

[4] Zhang Z Luo Y Shi M Li S Bao Y. EUS-FNA to diagnose a submucosal oropharyngeal carcinoma. Endosc Ultrasound. (2024) 13:273–5. doi: 10.1097/eus.0000000000000068, PMID: 39318750 PMC11419498

[5] Sung H Ferlay J Siegel RL Laversanne M Soerjomataram I Jemal A. Global cancer statistics 2020: GLOBOCAN estimates of incidence and mortality worldwide for 36 cancers in 185 countries. CA Cancer J Clin. (2021) 71:209–49. doi: 10.3322/caac.21660, PMID: 33538338

[6] Feng Y He C Liu C Shao B Wang D Wu P. Exploring the complexity and promise of tumor immunotherapy in drug development. Int J Mol Sci. (2024) 25:6444. doi: 10.3390/ijms25126444, PMID: 38928150 PMC11204037

[7] Nurgali K Jagoe RT Abalo R. Editorial: adverse effects of cancer chemotherapy: anything new to improve tolerance and reduce sequelae? Front Pharmacol. (2018) 9:245. doi: 10.3389/fphar.2018.00245, PMID: 29623040 PMC5874321

[8] Tavare AN Perry NJ Benzonana LL Takata M Ma D. Cancer recurrence after surgery: direct and indirect effects of anesthetic agents. Int J Cancer. (2012) 130:1237–50. doi: 10.1002/ijc.26448, PMID: 21935924

[9] Reck M Rodríguez-Abreu D Robinson AG Hui R Csőszi T Fülöp A. Five-year outcomes with pembrolizumab versus chemotherapy for metastatic non-small-cell lung cancer with PD-L1 tumor proportion score ≥ 50. J Clin Oncol. (2021) 39:2339–49. doi: 10.1200/jco.21.00174, PMID: 33872070 PMC8280089

[10] Du B Qin J Lin B Zhang J Li D Liu M. CAR-T therapy in solid tumors. Cancer Cell. (2025) 43:665–79. doi: 10.1016/j.ccell.2025.03.019, PMID: 40233718

[11] Choucair K Naqash AR Nebhan CA Nipp R Johnson DB Saeed A. Immune checkpoint inhibitors: the unexplored landscape of geriatric oncology. Oncologist. (2022) 27:778–89. doi: 10.1093/oncolo/oyac119, PMID: 35781739 PMC9438919

[12] Terry S Engelsen AST Buart S Elsayed WS Venkatesh GH Chouaib S. Hypoxia-driven intratumor heterogeneity and immune evasion. Cancer Lett. (2020) 492:1–10. doi: 10.1016/j.canlet.2020.07.004, PMID: 32712233

[13] Li Y Patel SP Roszik J Qin Y. Hypoxia-driven immunosuppressive metabolites in the tumor microenvironment: new approaches for combinational immunotherapy. Front Immunol. (2018) 9:1591. doi: 10.3389/fimmu.2018.01591, PMID: 30061885 PMC6054965

[14] Satge D. A tumor profile in primary immune deficiencies challenges the cancer immune surveillance concept. Front Immunol. (2018) 9:1149. doi: 10.3389/fimmu.2018.01149, PMID: 29881389 PMC5976747

[15] El Sissy C Marliot F Haicheur N Kirilovsky A Scripcariu D Lagorce-Pages C. Focus on the Immunoscore and its potential clinical implications. Ann Pathol. (2017) 37:29–38. doi: 10.1016/j.annpat.2016.12.010, PMID: 28161000

[16] Liu Z Liang Q Ren Y Guo C Ge X Wang L. Immunosenescence: molecular mechanisms and diseases. Signal Transduct Target Ther. (2023) 8:200. doi: 10.1038/s41392-023-01451-2, PMID: 37179335 PMC10182360

[17] Liu W Stachura P Xu HC Bhatia S Borkhardt A Lang PA. Senescent tumor CD8(+) T cells: mechanisms of induction and challenges to immunotherapy. Cancers (Basel). (2020) 12:2828. doi: 10.3390/cancers12102828, PMID: 33008037 PMC7601312

[18] Otegui N Houry M Arozarena I Serrano D Redin E Exposito F. Cancer cell-intrinsic alterations associated with an immunosuppressive tumor microenvironment and resistance to immunotherapy in lung cancer. Cancers (Basel). (2023) 15:3076. doi: 10.3390/cancers15123076, PMID: 37370686 PMC10295869

[19] Zhu S Luo Z Li X Han X Shi S Zhang T. Tumor-associated macrophages: role in tumorigenesis and immunotherapy implications. J Cancer. (2021) 12:54–64. doi: 10.7150/jca.49692, PMID: 33391402 PMC7738842

[20] Marcq E Siozopoulou V De Waele J van Audenaerde J Zwaenepoel K Santermans E. Prognostic and predictive aspects of the tumor immune microenvironment and immune checkpoints in Malignant pleural mesothelioma. Oncoimmunology. (2017) 6:e1261241. doi: 10.1080/2162402X.2016.1261241, PMID: 28197385 PMC5283621

[21] Anderson NR Minutolo NG Gill S Klichinsky M. Macrophage-based approaches for cancer immunotherapy. Cancer Res. (2021) 81:1201–8. doi: 10.1158/0008-5472.Can-20-2990, PMID: 33203697

[22] Williams CB Yeh ES Soloff AC. Tumor-associated macrophages: unwitting accomplices in breast cancer Malignancy. NPJ Breast Cancer. (2016) 2:15025–. doi: 10.1038/npjbcancer.2015.25, PMID: 26998515 PMC4794275

[23] Lin Y Xu J Lan H. Tumor-associated macrophages in tumor metastasis: biological roles and clinical therapeutic applications. J Hematol Oncol. (2019) 12:76. doi: 10.1186/s13045-019-0760-3, PMID: 31300030 PMC6626377

[24] Ralph SJ Reynolds MJ. Intratumoral pro-oxidants promote cancer immunotherapy by recruiting and reprogramming neutrophils to eliminate tumors. Cancer Immunol Immunother. (2023) 72:527–42. doi: 10.1007/s00262-022-03248-8, PMID: 36066649 PMC9446783

[25] Shimasaki N Jain A Campana D. NK cells for cancer immunotherapy. Nat Rev Drug Discov. (2020) 19:200–18. doi: 10.1038/s41573-019-0052-1, PMID: 31907401

[26] Kumar V Patel S Tcyganov E Gabrilovich DI. The nature of myeloid-derived suppressor cells in the tumor microenvironment. Trends Immunol. (2016) 37:208–20. doi: 10.1016/j.it.2016.01.004, PMID: 26858199 PMC4775398

[27] Lasser SA Ozbay Kurt FG Arkhypov I Utikal J Umansky V. Myeloid-derived suppressor cells in cancer and cancer therapy. Nat Rev Clin Oncol. (2024) 21:147–64. doi: 10.1038/s41571-023-00846-y, PMID: 38191922

[28] Oh K Lee OY Shon SY Nam O Ryu PM Seo MW. A mutual activation loop between breast cancer cells and myeloid-derived suppressor cells facilitates spontaneous metastasis through IL-6 trans-signaling in a murine model. Breast Cancer Res. (2013) 15:R79. doi: 10.1186/bcr3473, PMID: 24021059 PMC3979084

[29] Lu W Kang Y. Epithelial-mesenchymal plasticity in cancer progression and metastasis. Dev Cell. (2019) 49:361–74. doi: 10.1016/j.devcel.2019.04.010, PMID: 31063755 PMC6506183

[30] Garris CS Arlauckas SP Kohler RH Trefny MP Garren S Piot C. Successful anti-PD-1 cancer immunotherapy requires T cell-dendritic cell crosstalk involving the cytokines IFN-γ and IL-12. Immunity. (2018) 49:1148–1161.e1147. doi: 10.1016/j.immuni.2018.09.024, PMID: 30552023 PMC6301092

[31] de Mingo Pulido Á Gardner A Hiebler S Soliman H Rugo HS Krummel MF. TIM-3 regulates CD103(+) dendritic cell function and response to chemotherapy in breast cancer. Cancer Cell. (2018) 33:60–74.e66. doi: 10.1016/j.ccell.2017.11.019, PMID: 29316433 PMC5764109

[32] Salmon H Idoyaga J Rahman A Leboeuf M Remark R Jordan S. Expansion and activation of CD103(+) dendritic cell progenitors at the tumor site enhances tumor responses to therapeutic PD-L1 and BRAF inhibition. Immunity. (2016) 44:924–38. doi: 10.1016/j.immuni.2016.03.012, PMID: 27096321 PMC4980762

[33] Fang Z Meng Q Xu J Wang W Zhang B Liu J. Signaling pathways in cancer-associated fibroblasts: recent advances and future perspectives. Cancer Commun (Lond). (2023) 43:3–41. doi: 10.1002/cac2.12392, PMID: 36424360 PMC9859735

[34] Elyada E Bolisetty M Laise P Flynn WF Courtois ET Burkhart RA. Cross-species single-cell analysis of pancreatic ductal adenocarcinoma reveals antigen-presenting cancer-associated fibroblasts. Cancer Discov. (2019) 9:1102–23. doi: 10.1158/2159-8290.Cd-19-0094, PMID: 31197017 PMC6727976

[35] Morotti M Grimm AJ Hope HC Arnaud M Desbuisson M Rayroux N. PGE(2) inhibits TIL expansion by disrupting IL-2 signalling and mitochondrial function. Nature. (2024) 629:426–34. doi: 10.1038/s41586-024-07352-w, PMID: 38658764 PMC11078736

[36] Zou W Green DR. Beggars banquet: Metabolism in the tumor immune microenvironment and cancer therapy. Cell Metab. (2023) 35:1101–13. doi: 10.1016/j.cmet.2023.06.003, PMID: 37390822 PMC10527949

[37] Tan SN Hao J Ge J Yang Y Liu L Huang J. Regulatory T cells converted from Th1 cells in tumors suppress cancer immunity via CD39. J Exp Med. (2025) 222:e20240445. doi: 10.1084/jem.20240445, PMID: 39907686 PMC11797014

[38] Xiao X Lao XM Chen MM Liu RX Wei Y Ouyang FZ. PD-1hi identifies a novel regulatory B-cell population in human hepatoma that promotes disease progression. Cancer Discov. (2016) 6:546–59. doi: 10.1158/2159-8290.Cd-15-1408, PMID: 26928313

[39] Shang J Zha H Sun Y. Phenotypes, functions, and clinical relevance of regulatory B cells in cancer. Front Immunol. (2020) 11:582657. doi: 10.3389/fimmu.2020.582657, PMID: 33193391 PMC7649814

[40] Chen Z Zhang G Ren X Yao Z Zhou Q Ren X. Cross-talk between myeloid and B cells shapes the distinct microenvironments of primary and secondary liver cancer. Cancer Res. (2023) 83:3544–61. doi: 10.1158/0008-5472.Can-23-0193, PMID: 37352379

[41] Zannikou M Duffy JT Levine RN Seblani M Liu Q Presser A. IL15 modification enables CAR T cells to act as a dual targeting agent against tumor cells and myeloid-derived suppressor cells in GBM. J Immunother Cancer. (2023) 11:e006239. doi: 10.1136/jitc-2022-006239, PMID: 36759014 PMC9923337

[42] Cai M Huang X Huang X Ju D Zhu YZ Ye L. Research progress of interleukin-15 in cancer immunotherapy. Front Pharmacol. (2023) 14:1184703. doi: 10.3389/fphar.2023.1184703, PMID: 37251333 PMC10213988

[43] Ferrer I Alcántara S Ballabriga J Olivé M Blanco R Rivera R. Transforming growth factor-alpha (TGF-alpha) and epidermal growth factor-receptor (EGF-R) immunoreactivity in normal and pathologic brain. Prog Neurobiol. (1996) 49:99–123. doi: 10.1016/0301-0082(96)00009-3, PMID: 8844822

[44] Batlle E Massagué J. Transforming growth factor-β Signaling in immunity and cancer. Immunity. (2019) 50:924–40. doi: 10.1016/j.immuni.2019.03.024, PMID: 30995507 PMC7507121

[45] Schroder K Hertzog PJ Ravasi T Hume DA. Interferon-gamma: an overview of signals, mechanisms and functions. J Leukoc Biol. (2004) 75:163–89. doi: 10.1189/jlb.0603252, PMID: 14525967

[46] O’Brien RM Cannon A Reynolds JV Lysaght J Lynam-Lennon N. Complement in tumourigenesis and the response to cancer therapy. Cancers (Basel). (2021) 13:1209. doi: 10.3390/cancers13061209, PMID: 33802004 PMC7998562

[47] Kankeu Fonkoua LA Sirpilla O Sakemura R Siegler EL Kenderian SS. CAR T cell therapy and the tumor microenvironment: Current challenges and opportunities. Mol Ther Oncolytics. (2022) 25:69–77. doi: 10.1016/j.omto.2022.03.009, PMID: 35434273 PMC8980704

[48] Kuczek DE Larsen AMH Thorseth ML Carretta M Kalvisa A Siersbæk MS. Collagen density regulates the activity of tumor-infiltrating T cells. J Immunother Cancer. (2019) 7:68. doi: 10.1186/s40425-019-0556-6, PMID: 30867051 PMC6417085

[49] Zhou W Jia Y Liu Y Chen Y Zhao P. Tumor microenvironment-based stimuli-responsive nanoparticles for controlled release of drugs in cancer therapy. Pharmaceutics. (2022) 14:2346. doi: 10.3390/pharmaceutics14112346, PMID: 36365164 PMC9694300

[50] Luo Z Dai Y Gao H. Development and application of hyaluronic acid in tumor targeting drug delivery. Acta Pharm Sin B. (2019) 9:1099–112. doi: 10.1016/j.apsb.2019.06.004, PMID: 31867159 PMC6900560

[51] Ma T Guo WW Zhang M He W Dongzhi C Gongye X. Tumor-derived exosomal CCT6A serves as a matchmaker introducing chemokines to tumor-associated macrophages in pancreatic ductal adenocarcinoma. Cell Death Dis. (2025) 16:382. doi: 10.1038/s41419-025-07720-y, PMID: 40374617 PMC12081750

[52] Chen S Sun J Zhou H Lei H Zang D Chen J. New roles of tumor-derived exosomes in tumor microenvironment. Chin J Cancer Res. (2024) 36:151–66. doi: 10.21147/j.issn.1000-9604.2024.02.05, PMID: 38751437 PMC11090792

[53] Chen J Zhang G Wan Y Xia B Ni Q Shan S. Immune cell-derived exosomes as promising tools for cancer therapy. J Control Release. (2023) 364:508–28. doi: 10.1016/j.jconrel.2023.11.003, PMID: 37939852

[54] Yang C Dou R Wei C Liu K Shi D Zhang C. Tumor-derived exosomal microRNA-106b-5p activates EMT-cancer cell and M2-subtype TAM interaction to facilitate CRC metastasis. Mol Ther. (2021) 29:2088–107. doi: 10.1016/j.ymthe.2021.02.006, PMID: 33571679 PMC8178444

[55] Yu H Pan J Zheng S Cai D Luo A Xia Z. Hepatocellular carcinoma cell-derived exosomal miR-21-5p induces macrophage M2 polarization by targeting rhoB. Int J Mol Sci. (2023) 24:4593. doi: 10.3390/ijms24054593, PMID: 36902024 PMC10003272

[56] Zhang W Yan Y Peng J Thakur A Bai N Yang K. Decoding roles of exosomal lncRNAs in tumor-immune regulation and therapeutic potential. Cancers (Basel). (2022) 15:286. doi: 10.3390/cancers15010286, PMID: 36612282 PMC9818565

[57] You S Li S Zeng L Song J Li Z Li W. Lymphatic-localized Treg-mregDC crosstalk limits antigen trafficking and restrains anti-tumor immunity. Cancer Cell. (2024) 42:1415–1433.e1412. doi: 10.1016/j.ccell.2024.06.014, PMID: 39029466

[58] Pei S Deng X Yang R Wang H Shi JH Wang X. Age-related decline in CD8(+) tissue resident memory T cells compromises antitumor immunity. Nat Aging. (2024) 4:1828–44. doi: 10.1038/s43587-024-00746-5, PMID: 39592880

[59] Peralta RM Xie B Lontos K Nieves-Rosado H Spahr K Joshi S. Dysfunction of exhausted T cells is enforced by MCT11-mediated lactate metabolism. Nat Immunol. (2024) 25:2297–307. doi: 10.1038/s41590-024-01999-3, PMID: 39516648 PMC11588660

[60] Watson MJ Vignali PDA Mullett SJ Overacre-Delgoffe AE Peralta RM Grebinoski S. Metabolic support of tumour-infiltrating regulatory T cells by lactic acid. Nature. (2021) 591:645–51. doi: 10.1038/s41586-020-03045-2, PMID: 33589820 PMC7990682

[61] Wang J Liu C Wang P Liu Z Hu W Lv Z. Bioengineered tumor-derived extracellular vehicles suppressed colorectal cancer liver metastasis and bevacizumab resistance. Adv Sci (Weinh). (2025) 12:e2417714. doi: 10.1002/advs.202417714, PMID: 40397411 PMC12199312

[62] Wang R Song S Qin J Yoshimura K Peng F Chu Y. Evolution of immune and stromal cell states and ecotypes during gastric adenocarcinoma progression. Cancer Cell. (2023) 41:1407–1426.e1409. doi: 10.1016/j.ccell.2023.06.005, PMID: 37419119 PMC10528152

[63] Yasuda T Wang YA. Gastric cancer immunosuppressive microenvironment heterogeneity: implications for therapy development. Trends Cancer. (2024) 10:627–42. doi: 10.1016/j.trecan.2024.03.008, PMID: 38600020 PMC11292672

[64] van Elsas MJ Middelburg J Labrie C Roelands J Schaap G Sluijter M. Immunotherapy-activated T cells recruit and skew late-stage activated M1-like macrophages that are critical for therapeutic efficacy. Cancer Cell. (2024) 42:1032–1050.e1010. doi: 10.1016/j.ccell.2024.04.011, PMID: 38759656

[65] Ruf B Bruhns M Babaei S Kedei N Ma L Revsine M. Tumor-associated macrophages trigger MAIT cell dysfunction at the HCC invasive margin. Cell. (2023) 186:3686–3705.e3632. doi: 10.1016/j.cell.2023.07.026, PMID: 37595566 PMC10461130

[66] Wu L Yan J Bai Y Chen F Zou X Xu J. An invasive zone in human liver cancer identified by Stereo-seq promotes hepatocyte-tumor cell crosstalk, local immunosuppression and tumor progression. Cell Res. (2023) 33:585–603. doi: 10.1038/s41422-023-00831-1, PMID: 37337030 PMC10397313

[67] Qiu X Zhou T Li S Wu J Tang J Ma G. Spatial single-cell protein landscape reveals vimentin(high) macrophages as immune-suppressive in the microenvironment of hepatocellular carcinoma. Nat Cancer. (2024) 5:1557–78. doi: 10.1038/s43018-024-00824-y, PMID: 39327501

[68] Yin Y Feng W Chen J Chen X Wang G Wang S. Immunosuppressive tumor microenvironment in the progression, metastasis, and therapy of hepatocellular carcinoma: from bench to bedside. Exp Hematol Oncol. (2024) 13:72. doi: 10.1186/s40164-024-00539-x, PMID: 39085965 PMC11292955

[69] Guo J Huang X Dou L Yan M Shen T Tang W. Aging and aging-related diseases: from molecular mechanisms to interventions and treatments. Signal Transduct Target Ther. (2022) 7:391. doi: 10.1038/s41392-022-01251-0, PMID: 36522308 PMC9755275

[70] Yin F He Y Li J Gao Y. Immune cell senescence in autoimmunity: implications for disease pathogenesis and therapeutic targeting. Front Immunol. (2025) 16:1596686. doi: 10.3389/fimmu.2025.1596686, PMID: 40852730 PMC12367673

[71] Liu B Peng Z Zhang H Zhang N Liu Z Xia Z. Regulation of cellular senescence in tumor progression and therapeutic targeting: mechanisms and pathways. Mol Cancer. (2025) 24:106. doi: 10.1186/s12943-025-02284-z, PMID: 40170077 PMC11963325

[72] Faheem MM Seligson ND Ahmad SM Rasool RU Gandhi SG Bhagat M. Convergence of therapy-induced senescence (TIS) and EMT in multistep carcinogenesis: current opinions and emerging perspectives. Cell Death Discov. (2020) 6:51. doi: 10.1038/s41420-020-0286-z, PMID: 32566256 PMC7295779

[73] He Y Qiu Y Yang X Lu G Zhao SS. Remodeling of tumor microenvironment by cellular senescence and immunosenescence in cervical cancer. Semin Cancer Biol. (2024) 108:17–32. doi: 10.1016/j.semcancer.2024.11.002, PMID: 39586414

[74] Maggiorani D Le O Lisi V Landais S Moquin-Beaudry G Lavallée VP. Senescence drives immunotherapy resistance by inducing an immunosuppressive tumor microenvironment. Nat Commun. (2024) 15:2435. doi: 10.1038/s41467-024-46769-9, PMID: 38499573 PMC10948808

[75] Hwang HJ Lee YR Kang D Lee HC Seo HR Ryu JK. Endothelial cells under therapy-induced senescence secrete CXCL11, which increases aggressiveness of breast cancer cells. Cancer Lett. (2020) 490:100–10. doi: 10.1016/j.canlet.2020.06.019, PMID: 32659248

[76] Lanna A Henson SM Escors D Akbar AN. The kinase p38 activated by the metabolic regulator AMPK and scaffold TAB1 drives the senescence of human T cells. Nat Immunol. (2014) 15:965–72. doi: 10.1038/ni.2981, PMID: 25151490 PMC4190666

[77] Masters AR Hall A Bartley JM Keilich SR Lorenzo EC Jellison ER. Assessment of lymph node stromal cells as an underlying factor in age-related immune impairment. J Gerontol A Biol Sci Med Sci. (2019) 74:1734–43. doi: 10.1093/gerona/glz029, PMID: 30721932 PMC6777091

[78] Lian J Yue Y Yu W Zhang Y. Immunosenescence: a key player in cancer development. J Hematol Oncol. (2020) 13:151. doi: 10.1186/s13045-020-00986-z, PMID: 33168037 PMC7653700

[79] JH J DG M. The immune response against human cytomegalovirus links cellular to systemic senescence. Cells. (2020) 9:766. doi: 10.3390/cells9030766, PMID: 32245117 PMC7140628

[80] Wencker M Turchinovich G Di Marco Barros R Deban L Jandke A Cope A. Innate-like T cells straddle innate and adaptive immunity by altering antigen-receptor responsiveness. Nat Immunol. (2014) 15:80–7. doi: 10.1038/ni.2773, PMID: 24241693 PMC6485477

[81] Akagi J Baba H. Prognostic value of CD57(+) T lymphocytes in the peripheral blood of patients with advanced gastric cancer. Int J Clin Oncol. (2008) 13:528–35. doi: 10.1007/s10147-008-0789-8, PMID: 19093181

[82] Gergues M Bari R Koppisetti S Gosiewska A Kang L Hariri RJ. Senescence, NK cells, and cancer: navigating the crossroads of aging and disease. Front Immunol. (2025) 16:1565278. doi: 10.3389/fimmu.2025.1565278, PMID: 40255394 PMC12006071

[83] Lopes-Paciencia S Saint-Germain E Rowell MC Ruiz AF Kalegari P Ferbeyre G. The senescence-associated secretory phenotype and its regulation. Cytokine. (2019) 117:15–22. doi: 10.1016/j.cyto.2019.01.013, PMID: 30776684

[84] Puzianowska-Kuźnicka M Owczarz M Wieczorowska-Tobis K Nadrowski P Chudek J Slusarczyk P. Interleukin-6 and C-reactive protein, successful aging, and mortality: the PolSenior study. Immun Ageing. (2016) 13:21. doi: 10.1186/s12979-016-0076-x, PMID: 27274758 PMC4891873

[85] Ferrucci L Fabbri E. Inflammageing: chronic inflammation in ageing, cardiovascular disease, and frailty. Nat Rev Cardiol. (2018) 15:505–22. doi: 10.1038/s41569-018-0064-2, PMID: 30065258 PMC6146930

[86] Jha SK De Rubis G Devkota SR Zhang Y Adhikari R Jha LA. Cellular senescence in lung cancer: Molecular mechanisms and therapeutic interventions. Ageing Res Rev. (2024) 97:102315. doi: 10.1016/j.arr.2024.102315, PMID: 38679394

[87] Chibaya L Murphy KC DeMarco KD Gopalan S Liu H Parikh CN. EZH2 inhibition remodels the inflammatory senescence-associated secretory phenotype to potentiate pancreatic cancer immune surveillance. Nat Cancer. (2023) 4:872–92. doi: 10.1038/s43018-023-00553-8, PMID: 37142692 PMC10516132

[88] Heyn H Li N Ferreira HJ Moran S Pisano DG Gomez A. Distinct DNA methylomes of newborns and centenarians. Proc Natl Acad Sci U S A. (2012) 109:10522–7. doi: 10.1073/pnas.1120658109, PMID: 22689993 PMC3387108

[89] Brunner S Herndler-Brandstetter D Arnold CR Wiegers GJ Villunger A Hackl M. Upregulation of miR-24 is associated with a decreased DNA damage response upon etoposide treatment in highly differentiated CD8(+) T cells sensitizing them to apoptotic cell death. Aging Cell. (2012) 11:579–87. doi: 10.1111/j.1474-9726.2012.00819.x, PMID: 22435726 PMC3427896

[90] Huang L Zhang C Jiang A Lin A Zhu L Mou W. T-cell senescence in the tumor microenvironment. Cancer Immunol Res. (2025) 13:618–32. doi: 10.1158/2326-6066.Cir-24-0894, PMID: 40232041

[91] Henson SM Lanna A Riddell NE Franzese O Macaulay R Griffiths SJ. p38 signaling inhibits mTORC1-independent autophagy in senescent human CD8^+^ T cells. J Clin Invest. (2014) 124:4004–16. doi: 10.1172/jci75051, PMID: 25083993 PMC4151208

[92] Chi H. Regulation and function of mTOR signalling in T cell fate decisions. Nat Rev Immunol. (2012) 12:325–38. doi: 10.1038/nri3198, PMID: 22517423 PMC3417069

[93] Frauwirth KA Riley JL Harris MH Parry RV Rathmell JC Plas DR. The CD28 signaling pathway regulates glucose metabolism. Immunity. (2002) 16:769–77. doi: 10.1016/s1074-7613(02)00323-0, PMID: 12121659

[94] Angelin A Gil-de-Gómez L Dahiya S Jiao J Guo L Levine MH. Foxp3 reprograms T cell metabolism to function in low-glucose, high-lactate environments. Cell Metab. (2017) 25:1282–1293.e1287. doi: 10.1016/j.cmet.2016.12.018, PMID: 28416194 PMC5462872

[95] Zhang M Cui J Chen H Cheng Y Chen Q Zong F. Increased SOAT2 expression in aged regulatory T cells is associated with altered cholesterol metabolism and reduced anti-tumor immunity. Nat Commun. (2025) 16:630. doi: 10.1038/s41467-025-56002-w, PMID: 39805872 PMC11729894

[96] Ye J Huang X Hsueh EC Zhang Q Ma C Zhang Y. Human regulatory T cells induce T-lymphocyte senescence. Blood. (2012) 120:2021–31. doi: 10.1182/blood-2012-03-416040, PMID: 22723548 PMC3437594

[97] Liu X Mo W Ye J Li L Zhang Y Hsueh EC. Regulatory T cells trigger effector T cell DNA damage and senescence caused by metabolic competition. Nat Commun. (2018) 9:249. doi: 10.1038/s41467-017-02689-5, PMID: 29339767 PMC5770447

[98] Ye J Ma C Hsueh EC Eickhoff CS Zhang Y Varvares MA. Tumor-derived γδ regulatory T cells suppress innate and adaptive immunity through the induction of immunosenescence. J Immunol. (2013) 190:2403–14. doi: 10.4049/jimmunol.1202369, PMID: 23355732 PMC3578061

[99] Si F Liu X Tao Y Zhang Y Ma F Hsueh EC. Blocking senescence and tolerogenic function of dendritic cells induced by γδ Treg cells enhances tumor-specific immunity for cancer immunotherapy. J Immunother Cancer. (2024) 12:e008219. doi: 10.1136/jitc-2023-008219, PMID: 38580332 PMC11002396

[100] Hu Y Hu Q Li Y Lu L Xiang Z Yin Z. γδ T cells: origin and fate, subsets, diseases and immunotherapy. Signal Transduct Target Ther. (2023) 8:434. doi: 10.1038/s41392-023-01653-8, PMID: 37989744 PMC10663641

[101] Huang Y Xie Y Zhang Y Liu Z Jiang W Ye Y. Single-cell transcriptome reveals the reprogramming of immune microenvironment during the transition from MASH to HCC. Mol Cancer. (2025) 24:177. doi: 10.1186/s12943-025-02370-2, PMID: 40500691 PMC12153197

[102] Li L Liu X Sanders KL Edwards JL Ye J Si F. TLR8-mediated metabolic control of human treg function: A mechanistic target for cancer immunotherapy. Cell Metab. (2019) 29:103–123.e105. doi: 10.1016/j.cmet.2018.09.020, PMID: 30344014 PMC7050437

[103] Grassi F De Ponte Conti B. The P2X7 receptor in tumor immunity. Front Cell Dev Biol. (2021) 9:694831. doi: 10.3389/fcell.2021.694831, PMID: 34239877 PMC8258391

[104] Parish ST Kim S Sekhon RK Wu JE Kawakatsu Y Effros RB. Adenosine deaminase modulation of telomerase activity and replicative senescence in human CD8 T lymphocytes. J Immunol. (2010) 184:2847–54. doi: 10.4049/jimmunol.0903647, PMID: 20147632 PMC3772624

[105] Sheppard KA Fitz LJ Lee JM Benander C George JA Wooters J. PD-1 inhibits T-cell receptor induced phosphorylation of the ZAP70/CD3zeta signalosome and downstream signaling to PKCtheta. FEBS Lett. (2004) 574:37–41. doi: 10.1016/j.febslet.2004.07.083, PMID: 15358536

[106] Martínez-Zamudio RI Dewald HK Vasilopoulos T Gittens-Williams L Fitzgerald-Bocarsly P Herbig U. Senescence-associated β-galactosidase reveals the abundance of senescent CD8+ T cells in aging humans. Aging Cell. (2021) 20:e13344. doi: 10.1111/acel.13344, PMID: 33939265 PMC8135084

[107] Laphanuwat P Gomes DCO Akbar AN. Senescent T cells: Beneficial and detrimental roles. Immunol Rev. (2023) 316:160–75. doi: 10.1111/imr.13206, PMID: 37098109 PMC10952287

[108] Appay V Nixon DF Donahoe SM Gillespie GM Dong T King A. HIV-specific CD8(+) T cells produce antiviral cytokines but are impaired in cytolytic function. J Exp Med. (2000) 192:63–75. doi: 10.1084/jem.192.1.63, PMID: 10880527 PMC1887711

[109] Prieto LI Sturmlechner I Graves SI Zhang C Goplen NP Yi ES. Senescent alveolar macrophages promote early-stage lung tumorigenesis. Cancer Cell. (2023) 41:1261–1275.e1266. doi: 10.1016/j.ccell.2023.05.006, PMID: 37267954 PMC10524974

[110] DeNardo DG Ruffell B. Macrophages as regulators of tumour immunity and immunotherapy. Nat Rev Immunol. (2019) 19:369–82. doi: 10.1038/s41577-019-0127-6, PMID: 30718830 PMC7339861

[111] Wu T Dai Y. Tumor microenvironment and therapeutic response. Cancer Lett. (2017) 387:61–8. doi: 10.1016/j.canlet.2016.01.043, PMID: 26845449

[112] Haston S Gonzalez-Gualda E Morsli S Ge J Reen V Calderwood A. Clearance of senescent macrophages ameliorates tumorigenesis in KRAS-driven lung cancer. Cancer Cell. (2023) 41:1242–1260.e1246. doi: 10.1016/j.ccell.2023.05.004, PMID: 37267953

[113] Huntington ND Cursons J Rautela J. The cancer-natural killer cell immunity cycle. Nat Rev Cancer. (2020) 20:437–54. doi: 10.1038/s41568-020-0272-z, PMID: 32581320

[114] Maskalenko NA Zhigarev D Campbell KS. Harnessing natural killer cells for cancer immunotherapy: dispatching the first responders. Nat Rev Drug Discov. (2022) 21:559–77. doi: 10.1038/s41573-022-00413-7, PMID: 35314852 PMC10019065

[115] Muntasell A Rojo F Servitja S Rubio-Perez C Cabo M Tamborero D. NK cell infiltrates and HLA class I expression in primary HER2(+) breast cancer predict and uncouple pathological response and disease-free survival. Clin Cancer Res. (2019) 25:1535–45. doi: 10.1158/1078-0432.Ccr-18-2365, PMID: 30523021

[116] Kalathil SG Thanavala Y. Natural killer cells and T cells in hepatocellular carcinoma and viral hepatitis: current status and perspectives for future immunotherapeutic approaches. Cells. (2021) 10:1332. doi: 10.3390/cells10061332, PMID: 34071188 PMC8227136

[117] Liu X Li L Si F Huang L Zhao Y Zhang C. NK and NKT cells have distinct properties and functions in cancer. Oncogene. (2021) 40:4521–37. doi: 10.1038/s41388-021-01880-9, PMID: 34120141 PMC12416827

[118] Bancaro N Calì B Troiani M Elia AR Arzola RA Attanasio G. Apolipoprotein E induces pathogenic senescent-like myeloid cells in prostate cancer. Cancer Cell. (2023) 41:602–619.e611. doi: 10.1016/j.ccell.2023.02.004, PMID: 36868226

[119] Hernandez-Segura A de Jong TV Melov S Guryev V Campisi J Demaria M. Unmasking transcriptional heterogeneity in senescent cells. Curr Biol. (2017) 27:2652–2660.e2654. doi: 10.1016/j.cub.2017.07.033, PMID: 28844647 PMC5788810

[120] Gorgoulis V Adams PD Alimonti A Bennett DC Bischof O Bishop C. Cellular senescence: defining a path forward. Cell. (2019) 179:813–27. doi: 10.1016/j.cell.2019.10.005, PMID: 31675495

[121] Kadota T Fujita Y Yoshioka Y Araya J Kuwano K Ochiya T. Emerging role of extracellular vesicles as a senescence-associated secretory phenotype: Insights into the pathophysiology of lung diseases. Mol Aspects Med. (2018) 60:92–103. doi: 10.1016/j.mam.2017.11.005, PMID: 29146100

[122] Acosta JC Banito A Wuestefeld T Georgilis A Janich P Morton JP. A complex secretory program orchestrated by the inflammasome controls paracrine senescence. Nat Cell Biol. (2013) 15:978–90. doi: 10.1038/ncb2784, PMID: 23770676 PMC3732483

[123] Zhao B Wu B Feng N Zhang X Zhang X Wei Y. Aging microenvironment and antitumor immunity for geriatric oncology: the landscape and future implications. J Hematol Oncol. (2023) 16:28. doi: 10.1186/s13045-023-01426-4, PMID: 36945046 PMC10032017

[124] Krtolica A Parrinello S Lockett S Desprez PY Campisi J. Senescent fibroblasts promote epithelial cell growth and tumorigenesis: a link between cancer and aging. Proc Natl Acad Sci U S A. (2001) 98:12072–7. doi: 10.1073/pnas.211053698, PMID: 11593017 PMC59769

[125] Wang L Tang C Cao H Li K Pang X Zhong L. Activation of IL-8 via PI3K/Akt-dependent pathway is involved in leptin-mediated epithelial-mesenchymal transition in human breast cancer cells. Cancer Biol Ther. (2015) 16:1220–30. doi: 10.1080/15384047.2015.1056409, PMID: 26121010 PMC4622725

[126] Goulet CR Champagne A Bernard G Vandal D Chabaud S Pouliot F. Cancer-associated fibroblasts induce epithelial-mesenchymal transition of bladder cancer cells through paracrine IL-6 signalling. BMC Cancer. (2019) 19:137. doi: 10.1186/s12885-019-5353-6, PMID: 30744595 PMC6371428

[127] Waugh DJ Wilson C. The interleukin-8 pathway in cancer. Clin Cancer Res. (2008) 14:6735–41. doi: 10.1158/1078-0432.Ccr-07-4843, PMID: 18980965

[128] Ruhland MK Loza AJ Capietto AH Luo X Knolhoff BL Flanagan KC. Stromal senescence establishes an immunosuppressive microenvironment that drives tumorigenesis. Nat Commun. (2016) 7:11762. doi: 10.1038/ncomms11762, PMID: 27272654 PMC4899869

[129] Jiang M Chen J Zhang W Zhang R Ye Y Liu P. Interleukin-6 trans-signaling pathway promotes immunosuppressive myeloid-derived suppressor cells via suppression of suppressor of cytokine signaling 3 in breast cancer. Front Immunol. (2017) 8:1840. doi: 10.3389/fimmu.2017.01840, PMID: 29326716 PMC5736866

[130] Mao FY Kong H Zhao YL Peng LS Chen W Zhang JY. Increased tumor-infiltrating CD45RA(-)CCR7(-) regulatory T-cell subset with immunosuppressive properties foster gastric cancer progress. Cell Death Dis. (2017) 8:e3002. doi: 10.1038/cddis.2017.388, PMID: 28817117 PMC5596574

[131] Ning N Lu J Li Q Li M Cai Y Wang H. Single-sEV profiling identifies the TACSTD2 + sEV subpopulation as a factor of tumor susceptibility in the elderly. J Nanobiotechnology. (2024) 22:222. doi: 10.1186/s12951-024-02456-x, PMID: 38698420 PMC11067244

[132] Jakhar R Crasta K. Exosomes as emerging pro-tumorigenic mediators of the senescence-associated secretory phenotype. Int J Mol Sci. (2019) 20:2547. doi: 10.3390/ijms20102547, PMID: 31137607 PMC6566274

[133] Frasca D Diaz A Romero M Blomberg BB. Human peripheral late/exhausted memory B cells express a senescent-associated secretory phenotype and preferentially utilize metabolic signaling pathways. Exp Gerontol. (2017) 87:113–20. doi: 10.1016/j.exger.2016.12.001, PMID: 27931848

[134] Zhou R Zhou J Muhuitijiang B Tan W. Construction and experimental validation of a B cell senescence-related gene signature to evaluate prognosis and immunotherapeutic sensitivity in bladder cancer. Funct Integr Genomics. (2022) 23:3. doi: 10.1007/s10142-022-00936-7, PMID: 36527532

[135] Eggert T Wolter K Ji J Ma C Yevsa T Klotz S. Distinct functions of senescence-associated immune responses in liver tumor surveillance and tumor progression. Cancer Cell. (2016) 30:533–47. doi: 10.1016/j.ccell.2016.09.003, PMID: 27728804 PMC7789819

[136] Relecom A Merhi M Inchakalody V Uddin S Rinchai D Bedognetti D. Emerging dynamics pathways of response and resistance to PD-1 and CTLA-4 blockade: tackling uncertainty by confronting complexity. J Exp Clin Cancer Res. (2021) 40:74. doi: 10.1186/s13046-021-01872-3, PMID: 33602280 PMC7893879

[137] Li X Shao C Shi Y Han W. Lessons learned from the blockade of immune checkpoints in cancer immunotherapy. J Hematol Oncol. (2018) 11:31. doi: 10.1186/s13045-018-0578-4, PMID: 29482595 PMC6389077

[138] Vukadin S Khaznadar F Kizivat T Vcev A Smolic M. Molecular mechanisms of resistance to immune checkpoint inhibitors in melanoma treatment: an update. Biomedicines. (2021) 9:835. doi: 10.3390/biomedicines9070835, PMID: 34356899 PMC8301472

[139] Wang S Xie K Liu T. Cancer immunotherapies: from efficacy to resistance mechanisms - not only checkpoint matters. Front Immunol. (2021) 12:690112. doi: 10.3389/fimmu.2021.690112, PMID: 34367148 PMC8335396

[140] Sharpe AH Pauken KE. The diverse functions of the PD1 inhibitory pathway. Nat Rev Immunol. (2018) 18:153–67. doi: 10.1038/nri.2017.108, PMID: 28990585

[141] Hossen MM Ma Y Yin Z Xia Y Du J Huang JY. Current understanding of CTLA-4: from mechanism to autoimmune diseases. Front Immunol. (2023) 14:1198365. doi: 10.3389/fimmu.2023.1198365, PMID: 37497212 PMC10367421

[142] Dar HH Epperly MW Tyurin VA Amoscato AA Anthonymuthu TS Souryavong AB. P. aeruginosa augments irradiation injury via 15-lipoxygenase-catalyzed generation of 15-HpETE-PE and induction of theft-ferroptosis. JCI Insight. (2022) 7:e156013. doi: 10.1172/jci.insight.156013, PMID: 35041620 PMC8876480

[143] Feng YD Ye W Tian W Meng JR Zhang M Sun Y. Old targets, new strategy: Apigenin-7-O-β-d-(-6″-p-coumaroyl)-glucopyranoside prevents endothelial ferroptosis and alleviates intestinal ischemia-reperfusion injury through HO-1 and MAO-B inhibition. Free Radic Biol Med. (2022) 184:74–88. doi: 10.1016/j.freeradbiomed.2022.03.033, PMID: 35398494

[144] Kouo T Huang L Pucsek AB Cao M Solt S Armstrong T. Galectin-3 shapes antitumor immune responses by suppressing CD8+ T cells via LAG-3 and inhibiting expansion of plasmacytoid dendritic cells. Cancer Immunol Res. (2015) 3:412–23. doi: 10.1158/2326-6066.CIR-14-0150, PMID: 25691328 PMC4390508

[145] Wang J Sanmamed MF Datar I Su TT Ji L Sun J. Fibrinogen-like protein 1 is a major immune inhibitory ligand of LAG-3. Cell. (2019) 176:334–347 e312. doi: 10.1016/j.cell.2018.11.010, PMID: 30580966 PMC6365968

[146] Johnston RJ Comps-Agrar L Hackney J Yu X Huseni M Yang Y. The immunoreceptor TIGIT regulates antitumor and antiviral CD8(+) T cell effector function. Cancer Cell. (2014) 26:923–37. doi: 10.1016/j.ccell.2014.10.018, PMID: 25465800

[147] Liu S Zhang H Li M Hu D Li C Ge B. Recruitment of Grb2 and SHIP1 by the ITT-like motif of TIGIT suppresses granule polarization and cytotoxicity of NK cells. Cell Death Differ. (2013) 20:456–64. doi: 10.1038/cdd.2012.141, PMID: 23154388 PMC3569986

[148] Stanietsky N Simic H Arapovic J Toporik A Levy O Novik A. The interaction of TIGIT with PVR and PVRL2 inhibits human NK cell cytotoxicity. Proc Natl Acad Sci U S A. (2009) 106:17858–63. doi: 10.1073/pnas.0903474106, PMID: 19815499 PMC2764881

[149] Yu X Harden K Gonzalez LC Francesco M Chiang E Irving B. The surface protein TIGIT suppresses T cell activation by promoting the generation of mature immunoregulatory dendritic cells. Nat Immunol. (2009) 10:48–57. doi: 10.1038/ni.1674, PMID: 19011627

[150] Zhu C Anderson AC Schubart A Xiong H Imitola J Khoury SJ. The Tim-3 ligand galectin-9 negatively regulates T helper type 1 immunity. Nat Immunol. (2005) 6:1245–52. doi: 10.1038/ni1271, PMID: 16286920

[151] Rangachari M Zhu C Sakuishi K Xiao S Karman J Chen A. Bat3 promotes T cell responses and autoimmunity by repressing Tim-3–mediated cell death and exhaustion. Nat Med. (2012) 18:1394–400. doi: 10.1038/nm.2871, PMID: 22863785 PMC3491118

[152] Tawbi HA SChadendorf D Lipson EJ Ascierto PA Matamala L Castillo Gutiérrez E. Relatlimab and nivolumab versus nivolumab in untreated advanced melanoma. N Engl J Med. (2022) 386:24–34. doi: 10.1056/NEJMoa2109970, PMID: 34986285 PMC9844513

[153] Banta KL Xu X Chitre AS Au-Yeung A Takahashi C O’Gorman WE. Mechanistic convergence of the TIGIT and PD-1 inhibitory pathways necessitates co-blockade to optimize anti-tumor CD8(+) T cell responses. Immunity. (2022) 55:512–526.e519. doi: 10.1016/j.immuni.2022.02.005, PMID: 35263569 PMC9287124

[154] Curigliano G Gelderblom H Mach N Doi T Tai D Forde PM. Phase I/ib clinical trial of sabatolimab, an anti-TIM-3 antibody, alone and in combination with spartalizumab, an anti-PD-1 antibody, in advanced solid tumors. Clin Cancer Res. (2021) 27:3620–9. doi: 10.1158/1078-0432.Ccr-20-4746, PMID: 33883177

[155] Olson DJ Odunsi K. Adoptive cell therapy for nonhematologic solid tumors. J Clin Oncol. (2023) 41:3397–407. doi: 10.1200/jco.22.01618, PMID: 37104722

[156] Tran E Robbins PF Rosenberg SA. ‘Final common pathway’ of human cancer immunotherapy: targeting random somatic mutations. Nat Immunol. (2017) 18:255–62. doi: 10.1038/ni.3682, PMID: 28198830 PMC6295671

[157] Chamberlain CA Bennett EP Kverneland AH Svane IM Donia M Met O. Highly efficient PD-1-targeted CRISPR-Cas9 for tumor-infiltrating lymphocyte-based adoptive T cell therapy. Mol Ther Oncolytics. (2022) 24:417–28. doi: 10.1016/j.omto.2022.01.004, PMID: 35141398 PMC8807971

[158] Sadelain M Brentjens R Riviere I. The promise and potential pitfalls of chimeric antigen receptors. Curr Opin Immunol. (2009) 21:215–23. doi: 10.1016/j.coi.2009.02.009, PMID: 19327974 PMC5548385

[159] Seliger B Ritz U Ferrone S. Molecular mechanisms of HLA class I antigen abnormalities following viral infection and transformation. Int J Cancer. (2006) 118:129–38. doi: 10.1002/ijc.21312, PMID: 16003759

[160] Ho WY Blattman JN Dossett ML Yee C Greenberg PD. Adoptive immunotherapy: engineering T cell responses as biologic weapons for tumor mass destruction. Cancer Cell. (2003) 3:431–7. doi: 10.1016/s1535-6108(03)00113-2, PMID: 12781360

[161] Xin Q Chen Y Sun X Li R Wu Y Huang X. CAR-T therapy for ovarian cancer: Recent advances and future directions. Biochem Pharmacol. (2024) 226:116349. doi: 10.1016/j.bcp.2024.116349, PMID: 38852648

[162] Lu J Ma Y Li Q Xu Y Xue Y Xu S. CAR Macrophages: a promising novel immunotherapy for solid tumors and beyond. biomark Res. (2024) 12:86. doi: 10.1186/s40364-024-00637-2, PMID: 39175095 PMC11342599

[163] Zhao Q Jiang Y Xiang S Kaboli PJ Shen J Zhao Y. Engineered TCR-T cell immunotherapy in anticancer precision medicine: pros and cons. Front Immunol. (2021) 12:658753. doi: 10.3389/fimmu.2021.658753, PMID: 33859650 PMC8042275

[164] Segaliny AI Li G Kong L Ren C Chen X Wang JK. Functional TCR T cell screening using single-cell droplet microfluidics. Lab Chip. (2018) 18:3733–49. doi: 10.1039/c8lc00818c, PMID: 30397689 PMC6279597

[165] Davenport AJ Cross RS Watson KA Liao Y Shi W Prince HM. Chimeric antigen receptor T cells form nonclassical and potent immune synapses driving rapid cytotoxicity. Proc Natl Acad Sci U S A. (2018) 115:E2068–e2076. doi: 10.1073/pnas.1716266115, PMID: 29440406 PMC5834689

[166] Matsueda S Chen L Li H Yao H Yu F. Recent clinical researches and technological development in TIL therapy. Cancer Immunol Immunother. (2024) 73:232. doi: 10.1007/s00262-024-03793-4, PMID: 39264449 PMC11393248

[167] Fujiwara K Shigematsu K Tachibana M Okada N. Development and functional analysis of an anticancer T-cell medicine with immune checkpoint inhibitory ability. IUBMB Life. (2020) 72:1649–58. doi: 10.1002/iub.2280, PMID: 32255257

[168] Paul S Lal G. The molecular mechanism of natural killer cells function and its importance in cancer immunotherapy. Front Immunol. (2017) 8:1124. doi: 10.3389/fimmu.2017.01124, PMID: 28955340 PMC5601256

[169] Morse MA Gwin WR 3rd Mitchell DA. Vaccine therapies for cancer: then and now. Target Oncol. (2021) 16:121–52. doi: 10.1007/s11523-020-00788-w, PMID: 33512679 PMC7845582

[170] Zhang Z Lu M Qin Y Gao W Tao L Su W. Neoantigen: A new breakthrough in tumor immunotherapy. Front Immunol. (2021) 12:672356. doi: 10.3389/fimmu.2021.672356, PMID: 33936118 PMC8085349

[171] Blass E Ott PA. Advances in the development of personalized neoantigen-based therapeutic cancer vaccines. Nat Rev Clin Oncol. (2021) 18:215–29. doi: 10.1038/s41571-020-00460-2, PMID: 33473220 PMC7816749

[172] Huber F Arnaud M Stevenson BJ Michaux J Benedetti F Thevenet J. A comprehensive proteogenomic pipeline for neoantigen discovery to advance personalized cancer immunotherapy. Nat Biotechnol. (2024) 43:1360–72. doi: 10.1038/s41587-024-02420-y, PMID: 39394480 PMC12339364

[173] Ma W Pham B Li T. Cancer neoantigens as potential targets for immunotherapy. Clin Exp Metastasis. (2022) 39:51–60. doi: 10.1007/s10585-021-10091-1, PMID: 33950415 PMC8097110

[174] Braun DA Moranzoni G Chea V McGregor BA Blass E Tu CR. A personalized cancer vaccine to prevent the return of high-risk kidney cancer. Nature. (2025) 639:474–82. doi: 10.1038/d41586-025-00308-8, PMID: 39910358

[175] Kinkead HL Hopkins A Lutz E Wu AA Yarchoan M Cruz K. Combining STING-based neoantigen-targeted vaccine with checkpoint modulators enhances antitumor immunity in murine pancreatic cancer. JCI Insight. (2018) 3:e122857. doi: 10.1172/jci.insight.122857, PMID: 30333318 PMC6237485

[176] Hu Z Ott PA Wu CJ. Towards personalized, tumour-specific, therapeutic vaccines for cancer. Nat Rev Immunol. (2018) 18:168–82. doi: 10.1038/nri.2017.131, PMID: 29226910 PMC6508552

[177] León-Letelier RA Bonifaz LC Fuentes-Pananá EM. OMIC signatures to understand cancer immunosurveillance and immunoediting: Melanoma and immune cells interplay in immunotherapy. J Leukoc Biol. (2019) 105:915–33. doi: 10.1002/jlb.Mr0618-241rr, PMID: 30698862

[178] Baxevanis CN Perez SA. Cancer dormancy: A regulatory role for endogenous immunity in establishing and maintaining the tumor dormant state. Vaccines (Basel). (2015) 3:597–619. doi: 10.3390/vaccines3030597, PMID: 26350597 PMC4586469

[179] Mosely SI Prime JE Sainson RC Koopmann JO Wang DY Greenawalt DM. Rational selection of syngeneic preclinical tumor models for immunotherapeutic drug discovery. Cancer Immunol Res. (2017) 5:29–41. doi: 10.1158/2326-6066.CIR-16-0114, PMID: 27923825

[180] Zhang SW Wang H Ding XH Xiao YL Shao ZM You C. Bidirectional crosstalk between therapeutic cancer vaccines and the tumor microenvironment: Beyond tumor antigens. Fundam Res. (2023) 3:1005–24. doi: 10.1016/j.fmre.2022.03.009, PMID: 38933006 PMC11197801

[181] Aptsiauri N Garrido F. The challenges of HLA class I loss in cancer immunotherapy: facts and hopes. Clin Cancer Res. (2022) 28:5021–9. doi: 10.1158/1078-0432.Ccr-21-3501, PMID: 35861868

[182] Rodems TS Heninger E Stahlfeld CN Gilsdorf CS Carlson KN Kircher MR. Reversible epigenetic alterations regulate class I HLA loss in prostate cancer. Commun Biol. (2022) 5:897. doi: 10.1038/s42003-022-03843-6, PMID: 36050516 PMC9437063

[183] Wu Y Ju Q Qian B Zhang F Shi H. The effectiveness of PD-1 inhibitors in non-small cell lung cancer (NSCLC) patients of different ages. Oncotarget. (2018) 9:7942–8. doi: 10.18632/oncotarget.23678, PMID: 29487704 PMC5814271

[184] Huff WX Bam M Shireman JM Kwon JH Song L Newman S. Aging- and tumor-mediated increase in CD8(+)CD28(-) T cells might impose a strong barrier to success of immunotherapy in glioblastoma. Immunohorizons. (2021) 5:395–409. doi: 10.4049/immunohorizons.2100008, PMID: 34103370 PMC8591704

[185] Ferrara R Naigeon M Auclin E Duchemann B Cassard L Jouniaux JM. Circulating T-cell immunosenescence in patients with advanced non-small cell lung cancer treated with single-agent PD-1/PD-L1 inhibitors or platinum-based chemotherapy. Clin Cancer Res. (2021) 27:492–503. doi: 10.1158/1078-0432.Ccr-20-1420, PMID: 32887723

[186] Zhivaki D Kennedy SN Park J Boriello F Devant P Cao A. Correction of age-associated defects in dendritic cells enables CD4(+) T cells to eradicate tumors. Cell. (2024) 187:3888–3903.e3818. doi: 10.1016/j.cell.2024.05.026, PMID: 38870946 PMC11283364

[187] Sceneay J Goreczny GJ Wilson K Morrow S DeCristo MJ Ubellacker JM. Interferon signaling is diminished with age and is associated with immune checkpoint blockade efficacy in triple-negative breast cancer. Cancer Discov. (2019) 9:1208–27. doi: 10.1158/2159-8290.Cd-18-1454, PMID: 31217296 PMC11167954

[188] Sas K Szabó E Vécsei L. Mitochondria, oxidative stress and the kynurenine system, with a focus on ageing and neuroprotection. Molecules. (2018) 23:191. doi: 10.3390/molecules23010191, PMID: 29342113 PMC6017505

[189] Yan J Chen D Ye Z Zhu X Li X Jiao H. Molecular mechanisms and therapeutic significance of Tryptophan Metabolism and signaling in cancer. Mol Cancer. (2024) 23:241. doi: 10.1186/s12943-024-02164-y, PMID: 39472902 PMC11523861

[190] Muliaditan T Halim L Whilding LM Draper B Achkova DY Kausar F. Synergistic T cell signaling by 41BB and CD28 is optimally achieved by membrane proximal positioning within parallel chimeric antigen receptors. Cell Rep Med. (2021) 2:100457. doi: 10.1016/j.xcrm.2021.100457, PMID: 35028604 PMC8714859

[191] Bachiller M Perez-Amill L Battram AM Carné SC Najjar A Verhoeyen E. NK cells enhance CAR-T cell antitumor efficacy by enhancing immune/tumor cells cluster formation and improving CAR-T cell fitness. J Immunother Cancer. (2021) 9:e002866. doi: 10.1136/jitc-2021-002866, PMID: 34433634 PMC8388291

[192] Lorenzo-Luaces P Sanchez L Saavedra D Crombet T van der Elst W Alonso A. Identifying predictive biomarkers of CIMAvaxEGF success in non-small cell lung cancer patients. BMC Cancer. (2020) 20:772. doi: 10.1186/s12885-020-07284-4, PMID: 32807114 PMC7433036

[193] Ma L Chen C Zhao C Li T Ma L Jiang J. Targeting carnitine palmitoyl transferase 1A (CPT1A) induces ferroptosis and synergizes with immunotherapy in lung cancer. Signal Transduct Target Ther. (2024) 9:64. doi: 10.1038/s41392-024-01772-w, PMID: 38453925 PMC10920667

[194] Dianat-Moghadam H Mahari A Salahlou R Khalili M Azizi M Sadeghzadeh H. Immune evader cancer stem cells direct the perspective approaches to cancer immunotherapy. Stem Cell Res Ther. (2022) 13:150. doi: 10.1186/s13287-022-02829-9, PMID: 35395787 PMC8994338

[195] Yuan S Stewart KS Yang Y Abdusselamoglu MD Parigi SM Feinberg TY. Ras drives Malignancy through stem cell crosstalk with the microenvironment. Nature. (2022) 612:555–63. doi: 10.1038/s41586-022-05475-6, PMID: 36450983 PMC9750880

[196] Thompson HL Smithey MJ Surh CD Nikolich-Žugich J. Functional and homeostatic impact of age-related changes in lymph node stroma. Front Immunol. (2017) 8:706. doi: 10.3389/fimmu.2017.00706, PMID: 28659930 PMC5469916

[197] Li YR Zúñiga-Pflücker JC. Thymus aging and immune reconstitution, progresses and challenges. Semin Immunol. (2023) 70:101837. doi: 10.1016/j.smim.2023.101837, PMID: 37659170

[198] Pang WW Price EA Sahoo D Beerman I Maloney WJ Rossi DJ. Human bone marrow hematopoietic stem cells are increased in frequency and myeloid-biased with age. Proc Natl Acad Sci U S A. (2011) 108:20012–7. doi: 10.1073/pnas.1116110108, PMID: 22123971 PMC3250139

[199] Pereira BI Akbar AN. Convergence of innate and adaptive immunity during human aging. Front Immunol. (2016) 7:445. doi: 10.3389/fimmu.2016.00445, PMID: 27867379 PMC5095488

[200] Li Y Huang Q Zhou Y He M Chen J Gao Y. The clinicopathologic and prognostic significance of programmed cell death ligand 1 (PD-L1) expression in patients with prostate cancer: A systematic review and meta-analysis. Front Pharmacol. (2018) 9:1494. doi: 10.3389/fphar.2018.01494, PMID: 30733677 PMC6354218

[201] Yang X Jiang L Jin Y Li P Hou Y Yun J. PD-L1 expression in chinese patients with advanced non-small cell lung cancer (NSCLC): A multi-center retrospective observational study. J Cancer. (2021) 12:7390–8. doi: 10.7150/jca.63003, PMID: 35003359 PMC8734414

[202] Tchkonia T Zhu Y van Deursen J Campisi J Kirkland JL. Cellular senescence and the senescent secretory phenotype: therapeutic opportunities. J Clin Invest. (2013) 123:966–72. doi: 10.1172/jci64098, PMID: 23454759 PMC3582125

[203] Park MD Le Berichel J Hamon P Wilk CM Belabed M Yatim N. Hematopoietic aging promotes cancer by fueling IL-1α-driven emergency myelopoiesis. Science. (2024) 386:eadn0327. doi: 10.1126/science.adn0327, PMID: 39236155 PMC7616710

[204] Özkan A van den Bos F Mooijaart SP Slingerland M Kapiteijn E de Miranda N. Geriatric predictors of response and adverse events in older patients with cancer treated with immune checkpoint inhibitors: A systematic review. Crit Rev Oncol Hematol. (2024) 194:104259. doi: 10.1016/j.critrevonc.2024.104259, PMID: 38199430

[205] Cunha Júnior AD Bragagnoli AC Costa FO Carvalheira JBC. Repurposing metformin for the treatment of gastrointestinal cancer. World J Gastroenterol. (2021) 27:1883–904. doi: 10.3748/wjg.v27.i17.1883, PMID: 34007128 PMC8108031

[206] Turbitt WJ Buchta Rosean C Weber KS Norian LA. Obesity and CD8 T cell metabolism: Implications for anti-tumor immunity and cancer immunotherapy outcomes. Immunol Rev. (2020) 295:203–19. doi: 10.1111/imr.12849, PMID: 32157710 PMC7416819

[207] Sukumar M Liu J Ji Y Subramanian M Crompton JG Yu Z. Inhibiting glycolytic metabolism enhances CD8+ T cell memory and antitumor function. J Clin Invest. (2013) 123:4479–88. doi: 10.1172/jci69589, PMID: 24091329 PMC3784544

[208] Wang Y Huang T Gu J Lu L. Targeting the metabolism of tumor-infiltrating regulatory T cells. Trends Immunol. (2023) 44:598–612. doi: 10.1016/j.it.2023.06.001, PMID: 37442660

[209] Zhu Y Tchkonia T Fuhrmann-Stroissnigg H Dai HM Ling YY Stout MB. Identification of a novel senolytic agent, navitoclax, targeting the Bcl-2 family of anti-apoptotic factors. Aging Cell. (2016) 15:428–35. doi: 10.1111/acel.12445, PMID: 26711051 PMC4854923

[210] Chen Q Song S Wei S Liu B Honjo S Scott A. ABT-263 induces apoptosis and synergizes with chemotherapy by targeting stemness pathways in esophageal cancer. Oncotarget. (2015) 6:25883–96. doi: 10.18632/oncotarget.4540, PMID: 26317542 PMC4694873

[211] Triana-Martínez F Picallos-Rabina P Da Silva-Álvarez S Pietrocola F Llanos S Rodilla V. Identification and characterization of Cardiac Glycosides as senolytic compounds. Nat Commun. (2019) 10:4731. doi: 10.1038/s41467-019-12888-x, PMID: 31636264 PMC6803708

[212] Guerrero A Herranz N Sun B Wagner V Gallage S Guiho R. Cardiac glycosides are broad-spectrum senolytics. Nat Metab. (2019) 1:1074–88. doi: 10.1038/s42255-019-0122-z, PMID: 31799499 PMC6887543

[213] Amor C Feucht J Leibold J Ho YJ Zhu C Alonso-Curbelo D. Senolytic CAR T cells reverse senescence-associated pathologies. Nature. (2020) 583:127–32. doi: 10.1038/s41586-020-2403-9, PMID: 32555459 PMC7583560

[214] Schmitt CA Wang B Demaria M. Senescence and cancer - role and therapeutic opportunities. Nat Rev Clin Oncol. (2022) 19:619–36. doi: 10.1038/s41571-022-00668-4, PMID: 36045302 PMC9428886

[215] Moiseeva O Deschênes-Simard X St-Germain E Igelmann S Huot G Cadar AE. Metformin inhibits the senescence-associated secretory phenotype by interfering with IKK/NF-κB activation. Aging Cell. (2013) 12:489–98. doi: 10.1111/acel.12075, PMID: 23521863

[216] Laberge RM Sun Y Orjalo AV Patil CK Freund A Zhou L. MTOR regulates the pro-tumorigenic senescence-associated secretory phenotype by promoting IL1A translation. Nat Cell Biol. (2015) 17:1049–61. doi: 10.1038/ncb3195, PMID: 26147250 PMC4691706

[217] Correia-Melo C Birch J Fielder E Rahmatika D Taylor J Chapman J. Rapamycin improves healthspan but not inflammaging in nfκb1(-/-) mice. Aging Cell. (2019) 18:e12882. doi: 10.1111/acel.12882, PMID: 30468013 PMC6351839

[218] Ginés C Cuesta S Kireev R García C Rancan L Paredes SD. Protective effect of resveratrol against inflammation, oxidative stress and apoptosis in pancreas of aged SAMP8 mice. Exp Gerontol. (2017) 90:61–70. doi: 10.1016/j.exger.2017.01.021, PMID: 28130161

[219] Veeramachaneni R Yu W Newton JM Kemnade JO Skinner HD Sikora AG. Metformin generates profound alterations in systemic and tumor immunity with associated antitumor effects. J Immunother Cancer. (2021) 9:e002773. doi: 10.1136/jitc-2021-002773, PMID: 34230113 PMC8261884

[220] Ferrario A Merli M Basilico C Maffioli M Passamonti F. Siltuximab and hematologic Malignancies. A focus in non Hodgkin lymphoma. Expert Opin Investig Drugs. (2017) 26:367–73. doi: 10.1080/13543784.2017.1288213, PMID: 28140696

[221] Chen R Chen B. Siltuximab (CNTO 328): a promising option for human Malignancies. Drug Des Devel Ther. (2015) 9:3455–8. doi: 10.2147/dddt.S86438, PMID: 26170629 PMC4494175

[222] Engels P Szolek A Hörner S Syrigos GV Hebbel K Schmidtke M. Actionable heterogeneity of hepatocellular carcinoma therapy-induced senescence. Cancer Immunol Immunother. (2025) 74:207. doi: 10.1007/s00262-025-04060-w, PMID: 40374812 PMC12081809

[223] Chibaya L Snyder J Ruscetti M. Senescence and the tumor-immune landscape: Implications for cancer immunotherapy. Semin Cancer Biol. (2022) 86:827–45. doi: 10.1016/j.semcancer.2022.02.005, PMID: 35143990 PMC9357237

[224] Thompson JA Schneider BJ Brahmer J Zaid MA Achufusi A Armand P. NCCN guidelines^®^ Insights: management of immunotherapy-related toxicities, version 2.2024. J Natl Compr Canc Netw. (2024) 22:582–92. doi: 10.6004/jnccn.2024.0057, PMID: 39536465

[225] Wang B Hu S Teng Y Chen J Wang H Xu Y. Current advance of nanotechnology in diagnosis and treatment for Malignant tumors. Signal Transduct Target Ther. (2024) 9:200. doi: 10.1038/s41392-024-01889-y, PMID: 39128942 PMC11323968

[226] Zhang H Xu X Shou X Liao W Jin C Chen C. Senolytic therapy enabled by senescent cell-sensitive biomimetic melanin nano-senolytics. Adv Healthc Mater. (2024) 13:e2401085. doi: 10.1002/adhm.202401085, PMID: 38796738

[227] Chen C Guo Q Fu H Yu J Wang L Sun Y. Asynchronous blockade of PD-L1 and CD155 by polymeric nanoparticles inhibits triple-negative breast cancer progression and metastasis. Biomaterials. (2021) 275:120988. doi: 10.1016/j.biomaterials.2021.120988, PMID: 34186238

[228] Uto T Wang X Sato K Haraguchi M Akagi T Akashi M. Targeting of antigen to dendritic cells with poly(gamma-glutamic acid) nanoparticles induces antigen-specific humoral and cellular immunity. J Immunol. (2007) 178:2979–86. doi: 10.4049/jimmunol.178.5.2979, PMID: 17312143

[229] Cho NH Cheong TC Min JH Wu JH Lee SJ Kim D. A multifunctional core-shell nanoparticle for dendritic cell-based cancer immunotherapy. Nat Nanotechnol. (2011) 6:675–82. doi: 10.1038/nnano.2011.149, PMID: 21909083

[230] Chou PY Lin SY Wu YN Shen CY Sheu MT Ho HO. Glycosylation of OVA antigen-loaded PLGA nanoparticles enhances DC-targeting for cancer vaccination. J Control Release. (2022) 351:970–88. doi: 10.1016/j.jconrel.2022.10.002, PMID: 36220488

[231] Jiang Q Wang K Zhang X Ouyang B Liu H Pang Z. Platelet membrane-camouflaged magnetic nanoparticles for ferroptosis-enhanced cancer immunotherapy. Small. (2020) 16:e2001704. doi: 10.1002/smll.202001704, PMID: 32338436

[232] Goltsev Y Samusik N Kennedy-Darling J Bhate S Hale M Vazquez G. Deep profiling of mouse splenic architecture with CODEX multiplexed imaging. Cell. (2018) 174:968–981.e915. doi: 10.1016/j.cell.2018.07.010, PMID: 30078711 PMC6086938

[233] Lin JR Izar B Wang S Yapp C Mei S Shah PM. Highly multiplexed immunofluorescence imaging of human tissues and tumors using t-CyCIF and conventional optical microscopes. Elife. (2018) 7:e31657. doi: 10.7554/eLife.31657, PMID: 29993362 PMC6075866

[234] Giesen C Wang HA Schapiro D Zivanovic N Jacobs A Hattendorf B. Highly multiplexed imaging of tumor tissues with subcellular resolution by mass cytometry. Nat Methods. (2014) 11:417–22. doi: 10.1038/nmeth.2869, PMID: 24584193

[235] Terekhova M Swain A Bohacova P Aladyeva E Arthur L Laha A. Single-cell atlas of healthy human blood unveils age-related loss of NKG2C(+)GZMB(-)CD8(+) memory T cells and accumulation of type 2 memory T cells. Immunity. (2023) 56:2836–2854.e2839. doi: 10.1016/j.immuni.2023.10.013, PMID: 37963457

[236] Mogilenko DA Shpynov O Andhey PS Arthur L Swain A Esaulova E. Comprehensive profiling of an aging immune system reveals clonal GZMK(+) CD8(+) T cells as conserved hallmark of inflammaging. Immunity. (2021) 54:99–115.e112. doi: 10.1016/j.immuni.2020.11.005, PMID: 33271118

[237] Murciano-Goroff YR Warner AB Wolchok JD. The future of cancer immunotherapy: microenvironment-targeting combinations. Cell Res. (2020) 30:507–19. doi: 10.1038/s41422-020-0337-2, PMID: 32467593 PMC7264181

[238] Zhang Y Esmail A Mazzaferro V Abdelrahim M. Newest therapies for cholangiocarcinoma: an updated overview of approved treatments with transplant oncology vision. Cancers (Basel). (2022) 14:5074. doi: 10.3390/cancers14205074, PMID: 36291857 PMC9600404

[239] Rizvi NA Hellmann MD Snyder A Kvistborg P Makarov V Havel JJ. Cancer immunology. Mutational landscape determines sensitivity to PD-1 blockade in non-small cell lung cancer. Science. (2015) 348:124–8. doi: 10.1126/science.aaa1348, PMID: 25765070 PMC4993154

[240] Liu C Yang M Zhang D Chen M Zhu D. Clinical cancer immunotherapy: Current progress and prospects. Front Immunol. (2022) 13:961805. doi: 10.3389/fimmu.2022.961805, PMID: 36304470 PMC9592930

[241] Hodi FS O’Day SJ McDermott DF Weber RW Sosman JA Haanen JB. Improved survival with ipilimumab in patients with metastatic melanoma. N Engl J Med. (2010) 363:711–23. doi: 10.1056/NEJMoa1003466, PMID: 20525992 PMC3549297

[242] Vega Cano KS Marmolejo Castañeda DH Escrivá-de-Romaní S Saura C. Systemic therapy for HER2-positive metastatic breast cancer: current and future trends. Cancers (Basel). (2022) 15:51. doi: 10.3390/cancers15010051, PMID: 36612047 PMC9817525

[243] Xu J Mu S Wang Y Yu S Wang Z. Recent advances in immunotherapy and its combination therapies for advanced melanoma: a review. Front Oncol. (2024) 14:1400193. doi: 10.3389/fonc.2024.1400193, PMID: 39081713 PMC11286497

[244] Zhang P Liu X Gu Z Jiang Z Zhao S Song Y. Targeting TIGIT for cancer immunotherapy: recent advances and future directions. biomark Res. (2024) 12:7. doi: 10.1186/s40364-023-00543-z, PMID: 38229100 PMC10790541

[245] Rousseau A Parisi C Barlesi F. Anti-TIGIT therapies for solid tumors: a systematic review. ESMO Open. (2023) 8:101184. doi: 10.1016/j.esmoop.2023.101184, PMID: 36933320 PMC10030909

[246] Rezaei M Tan J Zeng C Li Y Ganjalikhani-Hakemi M. TIM-3 in leukemia; immune response and beyond. Front Oncol. (2021) 11:753677. doi: 10.3389/fonc.2021.753677, PMID: 34660319 PMC8514831

[247] Lica JJ Pradhan B Safi K Jakóbkiewicz-Banecka J Hellmann A. Promising therapeutic strategies for hematologic Malignancies: innovations and potential. Molecules. (2024) 29:4280. doi: 10.3390/molecules29174280, PMID: 39275127 PMC11397263

[248] Huang Y Fan H Ti H. Tumor microenvironment reprogramming by nanomedicine to enhance the effect of tumor immunotherapy. Asian J Pharm Sci. (2024) 19:100902. doi: 10.1016/j.ajps.2024.100902, PMID: 38595331 PMC11002556

[249] Betof Warner A Corrie PG Hamid O. Tumor-infiltrating lymphocyte therapy in melanoma: facts to the future. Clin Cancer Res. (2023) 29:1835–54. doi: 10.1158/1078-0432.Ccr-22-1922, PMID: 36485001 PMC10183807

[250] Parums DV. Editorial: first regulatory approval for adoptive cell therapy with autologous tumor-infiltrating lymphocytes (TILs) - lifileucel (Amtagvi). Med Sci Monit. (2024) 30:e944927. doi: 10.12659/msm.944927, PMID: 38689550 PMC11071689

[251] Filin IY Mayasin YP Kharisova CB Gorodilova AV Kitaeva KV Chulpanova DS. Cell immunotherapy against melanoma: clinical trials review. Int J Mol Sci. (2023) 24:2413. doi: 10.3390/ijms24032413, PMID: 36768737 PMC9916554

[252] Dong C Tan D Sun H Li Z Zhang L Zheng Y. Interleukin-12 delivery strategies and advances in tumor immunotherapy. Curr Issues Mol Biol. (2024) 46:11548–79. doi: 10.3390/cimb46100686, PMID: 39451566 PMC11506767

[253] Kang J Sun T Zhang Y. Immunotherapeutic progress and application of bispecific antibody in cancer. Front Immunol. (2022) 13:1020003. doi: 10.3389/fimmu.2022.1020003, PMID: 36341333 PMC9630604

[254] Wu HL Gong Y Ji P Xie YF Jiang YZ Liu GY. Targeting nucleotide metabolism: a promising approach to enhance cancer immunotherapy. J Hematol Oncol. (2022) 15:45. doi: 10.1186/s13045-022-01263-x, PMID: 35477416 PMC9044757

